# Tele-dentistry, its trends, scope, and future framework in oral medicine; a scoping review during January 1999 to December 2021

**DOI:** 10.1186/s13690-023-01128-w

**Published:** 2023-06-14

**Authors:** Fatemeh Niknam, Roxana Sharifian, Azadeh Bashiri, Maryam Mardani, Reza Akbari, Haitham Tuffaha, Loc Do, Peivand Bastani

**Affiliations:** 1 Department of Health Information Management, School of Health Management and Information Sciences, Student Research Committee, Health Human Resources Research Center, Shiraz University of Medical Sciences, Shiraz, Iran; 2 Department of Health Information Management, School of Health Management and Information Sciences, Health Human Resources Research Center, Shiraz University of Medical Sciences, Shiraz, Iran; 3 Oral and Dental Disease Research Center, School of Dentistry, Shiraz University of Medical Sciences, Shiraz, Iran; 4 Department of Oral & Maxillofacial Medicine, School of Dentistry, Oral and Dental Disease Research Center, Shiraz University of Medical Sciences, Shiraz, Iran; 5 Department of Computer Engineering and Information Technology, Shiraz University of Technology, Shiraz, Iran; 6 Centre for the Business and Economics of Health, Faculty of Business Economics and Law, The University of Queensland, Brisbane, Australia; 7 School of Dentistry, Faculty of Health and Behavioural Sciences, Oral Health Centre, The University of Queensland, Brisbane, Australia

**Keywords:** Tele-dentistry, Oral medicine, Facilitators, Barriers

## Abstract

**Background:**

Tele-dentistry has been increasingly used for different purposes of visit, consultation, triage, screening, and training in oral medicine. This study aims to determine the main facilitators, barriers, and participants` viewpoints of applying tele-dentistry in oral medicine and develop a framework indicating the input, process, output, and feedback.

**Method:**

This was a scoping review conducted in 2022 applying Arksey and O’Malley (2005) approach. Four databases including ISI web of science, PubMed, Scopus, and ProQuest were searched from January 1999 to December 2021. Inclusion criteria consisted of all original and non-original articles (reviews, editorials, letters, comments, and book chapters), and dissertations in English with a full text electronic file. Excel_2016_ was used for descriptive quantitative analysis and MAXQDA version 10 was applied for qualitative thematic analysis. A thematic framework was developed customizing the results of the review in a virtual mini expert panel.

**Results:**

Descriptive results show that among 59 included articles, 27 (46%) have addressed the various applications of tele-dentistry during COVID-19 pandemic in the field of oral medicine. From geographical distribution perspective, most of the papers were published in Brazil (*n* = 13)/ 22.03%, India (*n* = 7)/11.86% and USA (*n* = 6)/10.17%. Thematic analysis shows that seven main themes of “information”, “skill”, “human resource”, ‘technical”, “administrative’, ‘financial’, and ‘training and education’ are explored as facilitators. ‘Individual’, ‘environmental’, ‘organizational’, ‘regulation’, ‘clinical’, and ‘technical barriers’ are also identified as main barriers of tele-dentistry in oral medicine.

**Conclusion:**

According to the results for using tele-dentistry services in oral medicine, a diverse category of facilitators should be considered and at the same time, different barriers should be managed. Users` satisfaction and perceived usefulness of tele-dentistry as final outcomes can be increased considering the system`s feedback and applying facilitator incentives as well as decreasing the barriers.

**Supplementary Information:**

The online version contains supplementary material available at 10.1186/s13690-023-01128-w.

## Introduction

Tele-dentistry can be considered as the transmission of “Real-time” or “Store and forward” clinical information using electronic health records, digital imaging, photography, information and communications technology (ICT) and Internet to provide oral healthcare and dental services [1]. Tele-dentistry services are used in various fields of dentistry, one of which is oral medicine [2]. Oral medicine which is considered as one of the dentistry specialties dealing with diagnosis and nonsurgical management of oral mucosal and salivary gland disease, orofacial pain and, malignancies [1, 2] plays a substantial role in managing the patients with systemic diseases and comorbidities [3].

There are potential systemic implications and complexities for some oral conditions in the field of oral medicine [3]. To make accurate clinical decisions and timely diagnosis of these conditions, both additional diagnostic tests and the experienced clinicians are required [4, 5]. On this matter, some problems are posed including delayed diagnosis of oral cancers in underserved communities [6] due to the lack of oral medicine specialist, unexperienced community health workers and dentists, and their insufficient training to manage complex and complicated oral conditions [3, 7]. In addition, inability in managing patients’ condition may lead to increasing the rate of unnecessary referrals to oral medicine specialist [8, 9]. Such a great demand for visiting the specialists imposes the high transportation costs and a long waiting list on patients [10]. Such inconvenient circumstance has been intensified during COVID-19 pandemic when there was several challenges for patients and clinicians among them we can point to disruption and delay in regular visits for patients with chronic conditions [11, 12] and lack of patients` access to oral medicine services [12], as well as interruption or decline at dental students and oral medicine trainees` educational programs [13].

Tele-dentistry not only can be considered as a solution to handle the above problems [14], but also can provide an effective interaction between healthcare providers and patients for different purposes of visit [5, 13], consultation [13, 15], triage [16–18], screening [19, 20], and training [7, 21, 22]. Despite the advantages, it should not be overlooked that utilization and implementation of tele-dentistry applications in oral medicine can be faced with some limitations and challenges such as technological, financial, ethical, and legal problems [1, 23–27]. Therefore, awareness of the barriers and scrutiny of which to take appropriate actions play an essential role in the productivity of tele-dentistry [28].

Given that beyond outcomes such as the accuracy of tele-dentistry [6], to the best of our knowledge, there is limited evidence on applying tele-dentistry in the field of oral medicine. At the same time, to achieve a comprehensive identification of tele-dentistry applications in the area and develop applied interventions by oral health policy makers and specialists, it is important to determine the benefits and potential applications of tele-dentistry in oral medicine along with the facilitators, barriers, and the description of the participants` perception. So, the present scoping review was conducted to synthesize the evidence on the barriers and facilitators of applying tele-dentistry in the field of oral medicine as well as developing a framework of input, process, output, and feedback. The results of the scoping review can be served as a guide for oral health policy makers and administrators of the dentistry schools to achieve successful and effective implementation of tele-dentistry projects in the field of oral medicine.

## Method

This scoping review was conducted in accordance with Arksey and O’Malley's five-stage framework [29] and the JBI’s guideline [30] in 2022. The following six steps have been taken for this scoping review: identifying research questions, recognizing relevant studies, selecting relevant studies, charting the data, collating, summarizing, and reporting the results, and consultation as an optional step. The details in each step are described as follows.

### Identifying research questions

The main research question was “how to determine the benefits, potential applications and a framework of applying tele-dentistry in oral medicine?”. Four key objectives then were defined as follows:

- To identify characteristics of tele-dentistry studies in the field of oral medicine,
- To identify barriers of tele dentistry application in the field of oral medicine,
- To identify facilitators of tele-dentistry, use in the field of oral medicine,
- and to identify participants’ view about tele-dentistry in the field of oral medicine.

A research question of a scoping review should cover the population, the concept, and the context (PCC). Here, according to the research question, the population was defined as all the publications which consider tele-dentistry in the field of oral medicine. The concept included all the facilitators and barriers which help applying tele-dentistry in oral medicine or restricting the usage as well as the participants` views. And the context contained all the technical, infrastructural, clinical, and organizational factors related to the clinics and dentistry hospitals which apply tele-dentistry in oral medicine.

### Identifying relevant studies

In this step, keywords for the scoping review were selected following a preliminary review of the literature. We have searched four databases including ISI web of science, PubMed, Scopus, and ProQuest. The search duration was defined from January 1999 to December 2021. We selected 1999 as an initial year based on the results of a published systematic review [6]. Another reason for choosing this year to limit the search was that the most important and relevant field of study was retrieved in 1999. The inclusion criteria were all original articles, non-original articles consisting of reviews, editorials, letters, commentaries and book chapters, and dissertations. Moreover, all full-text of papers were in English-language. Systematic reviews and studies about remote screening routines in dentistry, teledentistry in oral radiology and tele-dentistry in oral and maxillofacial surgery were excluded. The logical operators, “OR” and “AND,” were used to increase the search sensitivity. The study keywords were chosen as a result of a preliminary review of the literature. The selected keywords, based on their relevant Medical Subject Headings (MeSH), have been searched in various databases. EndNote reference manager X8.1 (Clarivate Analytics, Philadelphia, PA, USA) was used to manage the retrieved reference and find duplicate references. Table1- Appendix shows the finalized search strategy of the scoping review.

### Study selection

**Fig. 1:**
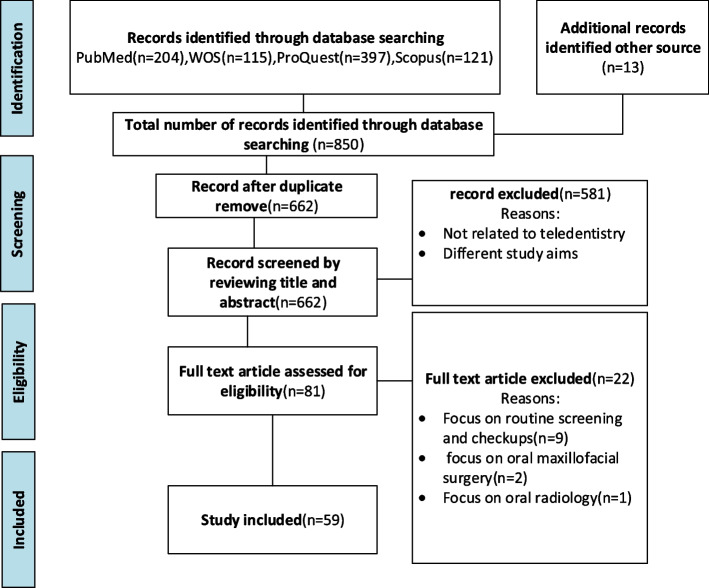
PRISMA flowchart; This diagram shows the systematic process followed to include literature in the scoping review on tele-dentistry in oral medicine (1991–2021)

After confirming the search strategy, the achieved articles via the systematic search of the four mentioned databases were reviewed first by the title and abstract and then by their full texts. All the process was conducted by two of the researchers separately and independently (FN and PB. Additional records from other sources were also searched through Google Scholar to complete the search,

For assuring the eligibility of the included full-texts, another researcher (MM) screened the full-text of the studies based on PCC, the review`s research question and objectives (RQ) and the inclusion and exclusion criteria. Figure 1 illustrates PRISMA Flow Diagram for the scoping review process [31], a narrative description of the search decision process along with the search decision flowchart.

### Charting the data

After selecting the final studies based on the desired inclusion and exclusion criteria, data related to the field of tele-dentistry in oral medicine were extracted and included in data extraction forms applying Microsoft Excel_2016_. The first author’s name, country of origin, year of publication, aim and type of the study, study setting, study population, methodology, main findings, and implications were extracted and charted in the data extraction form. This charting process were implemented simultaneously and jointly by two of the research team`s members (FN and PB) [30]. The chart distinctly shows the characteristics of the included studies according to the data of the extraction form (Table2- Appendix).

### Collating, summarizing, and reporting the results

At this step, two researchers (FN and PB) independently integrated and summarized the texts to answer the study question. To categorize and summarize the data, the thematic analysis was used via the following steps [32]:

- *Familiarization*: the extracted data from the full text of the included papers were read several times to become familiar with the collected data.
- *Initial coding:* the extracted data were arranged into table of initial codes with appropriate labels. Via this second step, similar meaningful units were given the same codes.
- *Finding themes*: the third step was applied to integrate the initial codes into final codes. The inductive and deductive approach were used to categorize the initial codes to the final ones and the final codes to the sub-themes and main themes.
- *Reviewing themes*: the fourth step consisted of two stages: reviewing the themes, including the coded summaries to determine whether they are valid for the original data set, and refining the themes to make them more accurate and a better representative of the data.
- *Defining and naming the themes*: through the fifth step, the themes were named according to their definition considering that they provide a comprehensive representation of the main concepts. And finally, the themes were tabulated and reported the facilitators, barriers, and participant perception of applying tele-dentistry in oral medicine.

Excel_2016_ was used for descriptive quantitative analysis and MAXQDA version 10 was applied for qualitative thematic analysis. MAXQDA is a data analysis software which can be used for content analysis. For this purpose, the meaningful units extracted from the included articles were imported from Excel to "Document System" component of the MAXQDA software. Then, by using the component "Document Browser", a section of the text was dedicated to the code via selecting the function of "Code with new code" from the context menu. A dialogue box has been opened to define new codes in the top row of which a code name has been entered. Afterwards, the code categorization process was carried out by assigning categories.

### Consultation (optional)

To be sure of the rigorous and trustworthiness [33] of the findings and the appropriate description of the thematic map, the final tabulated themes synthesized from the included articles accompanied with the initial thematic map determined by the research team were discussed in a virtual mini expert panel including four well experienced and knowledgeable participants in digital health in dentistry.

## Results

Results of the study are presented in two parts: first, the description of the characteristics of the included studies and then the results of the thematic analysis to identify facilitators, barriers, and participants` view and the system framework.

### Part 1: Characteristics of the included studies

**Fig. 2:**
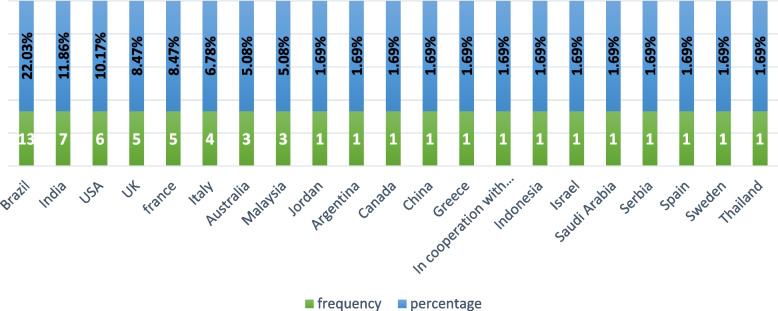
Distribution of tele-dentistry articles according to the studies` place,* (scoping review 1991–2021)*

A final selection of 59 studies were included in the scoping review. Figure 1 illustrates PRISMA Flow Diagram for the scoping review process. Out of 59 articles, 23(38.98%) articles were published in 2021. Table 2-Appendix shows the characteristics of these articles. Also, 27 (46%) out of 59 publications have addressed the various applications of tele-dentistry during COVID-19 pandemic in the field of oral medicine. From geographical distribution perspective, most of the papers were published in Brazil (*n* = 13)/22.03%, India (*n* = 7)/11.86%, USA(*n* = 6)/10.17%, UK (*n* = 5)/8.47% and France (*n* = 5)/8.47%. Figure 2 illustrates the distribution of the included articles according to their country.

**Fig. 3:**
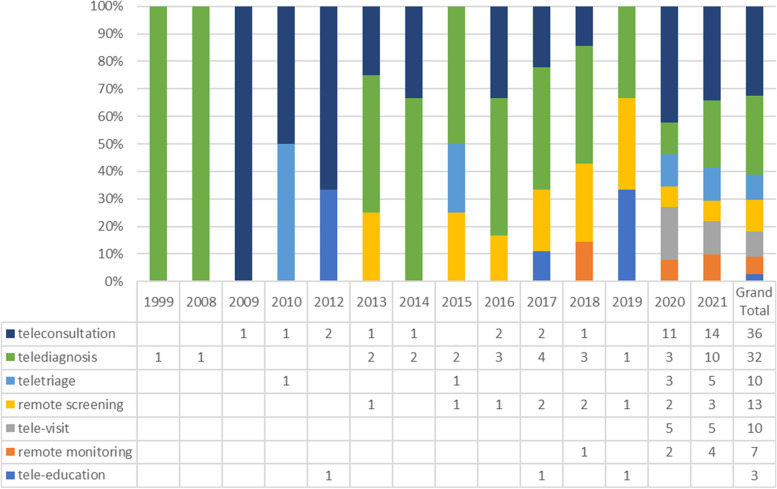
Distribution of tele-dentistry application type according to the studies publication year, *(scoping review 1991–2021).* Note: Each study includes more than one type of tele-dentistry application

Other descriptive results show that there are seven categories of different types of tele-dentistry applications. Figures 3 illustrates that tele-consultation and tele-diagnosis were used more than others. In particular, teleconsultation has been used more between 2020–2021. In addition, tele-consultation was applied to manage patients with oral medicine conditions, oral medicine referrals, assess, and follow up of patient, and education. Furthermore, the focus on the concepts of tele-triage, remote screening, tele-visit and telemonitoring has increased over the past two years (Fig. 3).

### Part 2: Thematic results

The results of the thematic analysis are divided in three following sections: facilitators and barriers of applying tele-dentistry in oral medicine, Participants’ view and developing the framework.

### Facilitators and barriers

Facilitators of tele-dentistry were categorized into seven themes, including information, skill, human resource, technical, administrative, financial, and training and education. Table 1 tabulates these seven facilitators accompanied with their sub-facilitators, item indications and descriptions.

**Table 1:** Facilitators and Barriers of applying tele-dentistry in oral medicine *(scoping review 1991–2021)*

Facilitators		Barriers	
**Main theme**	**Sub-theme**	**Main theme**	**Sub-theme**
**Information**	Applying patient information management tools Ex: EHR^*^ [7], Open MRS^*^ [34]	**Individual**	Behavioral barriers [4, 19, 23, 35–37]
	Following data quality criteria Ex: Data completeness [5, 10, 24], Data accuracy [5, 10, 24, 36],Timeliness of data [38]		Lack of experience and skill [11, 21, 25, 39]
**Skill**	Communication skills [5]		Human error [8, 24]
	Clinical skill [5, 25] Ex: Clinical reasoning [5], Differential diagnosis construction [5]	**Environmental**	Inappropriate environmental conditions [1, 11, 12, 23, 40, 41]
	Documentation skill [5]	**Technical**	Integration problems [3]
	Participants digital skill [11]		Internet bandwidth problems [1, 23, 24]
**Human resource**	Use of experienced clinicians [5, 36]		Problems in data storing [3, 23, 24]
	Using two remote clinicians and consultant [42]		Security and confidentiality problems [10, 26, 40]
	Using a trained assistant to record videos and photos [43]		Photo quality problems [20, 24]
**Technical**	Cloud-based platform [44]		Problems in real time interpretation [35, 45]
	Photography with smartphone [26, 38, 40, 46, 47]		Accessibility problems [7]
	Photography with intra oral camera [19, 39, 48]	**Regulation**	legal and ethical issues [41]
	Photography by intra oral camera with fluorescent aids [43, 48]		Prohibition of using some applications [20]
	Video recording [43, 49, 50]	**Organizational**	Human resource problems [1, 23]
	Video conferences [7, 12, 51]		Administrative Challenges [23]
	Access to affordable technologies [12, 20, 34, 45]		Financial Barriers [1, 10]
	User-friendly technologies [26, 45]		Lack of guidelines [1, 39]
	Wide penetration of the smartphone [11, 34]		Lack of time management [23, 24]
	Social media applications [20, 40, 52]	**Clinical**	Problems in Patient examination [11, 13, 35, 53]
	Photo transmission speed between onsite and remote health provider [38]		Making diagnosis problems [23, 26, 45]
	Digital pathology [50, 54]		Problems in performing treatment plan [55]
**Financial**	Reimbursement [7]		
	Free of charge tele medicine services [44]		
**Training and education**	Providing a guideline for taking a photo [36, 39]		
	Providing adequate training [26]		
	Training (healthcare worker/ health aids) [26]		
	Training (patients) [11]		
**Administrative**	collaboration and coordination between remote and hub sites [43] Ex: Coordination before tele-consulting by SMS^*^ and phone call [5], Collection of patient clinical information before the appointment [5], Set adequate time for the appointment [5], Collaboration between healthcare providers [5]		
	Involvement of local authorities [23]		

^*^EHR Electronic health record, *OpenMRS* Open Medical Record System, *SMS* Short message service

Similarly, the barriers in applying tele-dentistry in the field of oral medicine are tabulated in Table 1. These barriers have been classified into six domains individual and environmental, organizational, regulation, clinical, and technical barriers. As Table 1 implies most of these barriers are explored in the categories of individual barriers from the patients` side and the technical barriers of applying tele-dentistry in oral medicine. Additional explanations regarding barriers are also described in Appendix 2.

### Participants’ view

Two main concepts of satisfaction and perceived usefulness of the technology were analyzed from the participants’ view.

According to the thematic analysis of the included literature, participants who apply tele-dentistry services in the field of oral medicine include healthcare providers, healthcare systems and healthcare seekers. These participants clarify some reasons for satisfaction with their experiences of applying tele-dentistry, such as access to care during the COVID-19 pandemic [15, 56], providing oral medicine services in rural areas [15], communication between patients and doctors [15], the usability of tele-dentistry services [15, 57], quality in data communication [25], high-quality internet connection [40], and high-quality digital images with medical and dental information to facilitate diagnosis of oral mucosal disease [25]. They also provide some reasons for dissatisfaction with their experience in applying tele-dentistry, for example inadequate information [35], Lack of physical examination by a doctor [15] and not being involved in the care and treatment process during the consultation as much as they wanted [35].

From the aspect of usefulness perceived on tele-dentistry services by the participants, as Table 2 demonstrates, 14 domains are explored including time, access to oral medicine services, communication between dental clinicians, referrals, triage, travel, cost, education and training, health crisis, surveillance, quality of care, patient management and monitoring, and patient empowerment. Each of these domains include some implications that are synthesized and reported as items in Table 2.

**Table 2:** Categorizations of usefulness perceived on tele-dentistry services by participants* (scoping review 1991–2021)*

Domains	Items
**Time**	Save time (for patients/clinicians/staff) [11, 21, 24, 25, 58] Reduce the waiting time [11, 26, 58] Minimization of doctor’s delay [58]
**Access to oral medicine services**	Increase patient access to oral medicine services [5, 7, 18, 51, 58] A convenient way to access Oral Medicine services. [1, 3, 7, 51, 59] A convenient way for disabled patients in the remote population. [21] Improved access to remote specialists for unassisted populations [40] Facilitate patient–professional communication [18, 24] Continuous clinic visits and care [5, 7, 45] Improve access to preventative and diagnostic care for remote communities [7, 19, 27]
**Communication between dental clinicians**	Rapid access to specialist opinions for the general practitioners [58, 60] Practical means of communication between dental clinicians [45] Optimizing the use of specialists’ skill in underserved area [8] Access specialized knowledge globally through experts` integration [1] Access to specialist (dental hygienist located in a remote or underserved area/primary healthcare) [7, 57] Improve access to specialists (confirming diagnosis/detecting oral lesions/formulating treatment plan) [7, 27] Communication (share uncertainties, complexity of cases, experience and doing the best for the patient) [7, 60] Giving advice of secondary healthcare staff to primary healthcare staff base on appropriate information [45] Reduce isolation of practitioners through contact with peers and specialists [7] Management/diagnosis of oral lesions by remote support of specialists [7, 40] Allowing clinicians to treat the patients in an informed manner [8]
**Referrals**	Streamlining referral of patients [19, 61] Reduction of unnecessary referrals [8, 9, 19, 24, 40, 58] Aid the referral pathway from primary to secondary care [20] Ensuring timely referral [24] Suitable for management of referrals for older dependent adults with oral mucosal disease [25]
**Travel**	Reduce unnecessary traveling time and cost [7, 19, 24] No need to move from home [11] Environmental benefits due to reducing the travel distance [7, 51, 58]
**Quality of care**	Improved quality of care [7, 57]
**Patient empowerment**	Increasing patient’s awareness [5, 41] Self-monitoring [5] Risk-factor modification [5] Improves self-oral care [24] Triaging of patient through tele-consultation [24]
**Triage**	Improve the efficiency of specialty triaging [1] Ability to prioritize patient's medical needs [13] Effective triage of patients who need emergency clinical attention [1, 13, 18] Avoiding unnecessary clinical visits [10] Reduce the congestion at the hospital [19]
**Cost**	An economical method of preoperative assessment when patient transport is difficult or expensive [8, 58] Cost-effective way to organize healthcare [58] Saving the costs of referral [60] Reduce cost of oral health maintenance through shared resources [7] Suitable for elderly who avoid hospital-based treatment due to travel costs [25] Reduce cost of oral health maintenance through shared resources [7]
**Education and Training**	Learning opportunities for apprentices (dentists/dental students/assistants) [5, 7, 24] Collaboration to exchange experiences [7] Implementation of the treatment plan under the guidance of the specialist [7] Provide multipoint interactive continuing education courses [7] Multicenter treatment planning conferences [7] Inter-residency case reviews with community dentists at remote sites [7] Providing long-distance interactive training to local therapists at remote area [7] Originate multiple providers virtual care groups to provide distended clinical training [7] Improved access to specialists for clinical training [7] Improve the knowledge required for better oral cancer detection by distance learning courses [37] Create patient awareness of the harmful risk factor in oral cancer [24] Facilitate patient education about self-care [7] Provide a way to deliver long-distance clinical training and continuing education [7]
**Health crisis**	Ability to continue clinical education of oral medicine trainees during COVID-19 [13] Visits and ongoing care during COVID-19 pandemic [13] Convenience way to access to oral medicine during COVID-19 pandemic [22, 41, 62] Good option for advice or follow-up during COVID-19 [12, 46] Preparing for future health crises [41] Following the infection control protocols with tele-consultation prior to a face-to-face visit [62] Prioritize higher-risk patient while avoiding face-to-face contact during COVID-19 [17] Reducing unnecessary hospital visits during COVID-19 pandemic [17, 63] Monitor oral medical emergencies [63] Alleviate patient's anxiety related to delays in scheduling their office visit in COVID-19 [13] Provide clinical and supportive care to patients with oral diseases during pandemic [16] Management of oral medicine emergencies [12] Provide reassurance [12] Providing multidisciplinary care (group video calls) for patients who require the same [12] Prescription of routine blood tests via video conferencing software [12] Prevent increased morbidity of various dental and oral diseases due to delayed treatment during COVID-19 [15]
**Empowerment of local resources**	Enabling of primary care facilities for specialized diagnosis and treatment [10, 24] Provide direct support for a dental hygienist located in a remote area [7] Providing more accessible dental care and education by hygienists [7] Providing less expensive preventive dental care and education by hygienists [7] Support for remote early detection of oral cancer in resource-limited settings [23] Identifying oral lesions at primary level using m-Health and onsite/remote oral medicine specialist diagnosis [24] Large impact on optimizing resource utilization around specialty care [8]
**Surveillance**	Provide geo-marking for high-risk group by aiding in surveillance [24] Community screening and follow-up of patients [26] Early detection of oral cancer, particularly in low-resource setting [19, 21] Management of oral cancer and regular screening [62] Continued early diagnosis [59] Prevention of oral and pharyngeal cancer [59] Long term improving of oral cancer survival rates [34] Reduce the deficiencies in traditional screening methods by integrating health and technology [24]
**Patient management and monitoring**	Management of patients with oral mucosal disease [25] An efficient/well accepted approach of managing patients with chronic oral mucosal diseases [45, 56, 60] Follow-up during treatment by fixed and scheduled rounds [58] Integrate clinical setting for patient management [26] Facilitating diagnosis and treatment plane [60] Elderly patients’ management [25] Distant supervision/consultations of elderly patient [25] Increasing patient monitoring [41] Provision of supportive care for patients with oral mucosal conditions [13]

### Framework development

**Fig. 4:**
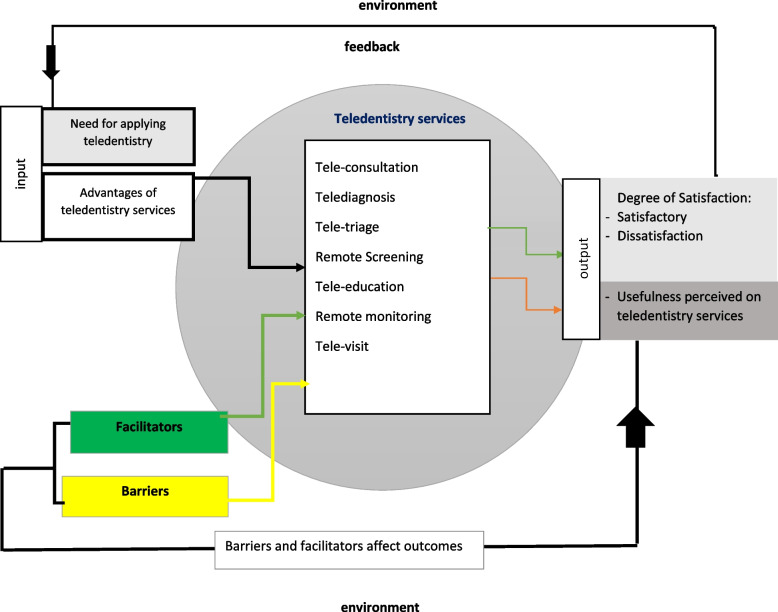
The framework of facilitators, barriers, and outcome for applying tele-dentistry in oral medicine,* (scoping review 1991–2021)*

The framework developed according to the main themes and using a general system model that includes input, process, and output. In other words, the results were modelled using a simple system analysis model known as IPO (input- process-output) [64].The need for applying tele-dentistry services and usefulness perceived or advantages of the tele-dentistry services by the users serve as input. In the process of using tele-dentistry services, there are some barriers and facilitators which could influence the process and ultimately the outputs. Outputs or outcomes such as user satisfaction and perceived usefulness are linked to feedback and used as a starting point for tele-dentistry services. In addition, according to participants’ view, these services were found to be useful in 14 domains and satisfactory, which has been affected by the application of facilitators. At the same time, negative outcomes such as dissatisfaction with services are emerging because of the probable barriers and limitations (Fig. 4).

## Discussion

The present scoping review synthesized the main facilitators, barriers, participants` view, and a framework indicating facilitators, barriers, and outcome for applying tele-dentistry in oral medicine.

According to the present descriptive results, tele-consultation and tele-diagnosis were used more than others. In particular, teleconsultation has been used more between 2020–2021.This high rate of usage of teleconsultation can be attributed to the COVID-19 pandemic, which has led to a rapid and sudden shift from traditional in-person consultations to the use of tele-consultations in providing health care [13, 35]. In particular, tele-dentistry enables dentists to use telecommunications to share clinical information and images remotely, which facilitates dental consultations related to diagnosis and treatment planning [65]. In addition, the nature, complexity, and some implications with oral medicine conditions could be among the reasons of this higher rate of usage [3]. Furthermore, to make final diagnosis decisions on oral medicine conditions, consult with oral medicine specialists [7, 27] will be necessary. According to the evidence, most dental practitioners cannot distinguish the correct diagnostic features of oral infections, especially fungal and viral infections without a consult with oral medicine specialist [65].

### Facilitators

According to the present thematic results, tele-dentistry facilitators were classified into seven groups. Most of the studies have concentrated on technical, informational and skill facilitators, respectively. In terms of technical facilitators, one of the infrastructures needed to implement tele-dentistry services is the technical aspect [66]. Using of social networks as a facilitator stem from their benefits. To illustrate, WhatsApp and similar applications are one of the strategies to facilitate daily patient-provider interactions and to accelerate clinical communication between oral health professionals [65]. In addition, their capabilities such as photo sharing, video chat, and messaging can be used in various aspects including screening, consultation, visits, and diagnosis [13, 20, 44, 65]. Furthermore, user access to technology has been one of the main facilitators identified in the studies. Indeed, elements such as using economical [12, 20, 34, 63] and user-friendly [26, 45] technologies and high level of mobile and internet penetration [11, 34] play a significant role in people`s access to tele-dentistry services. In terms of using affordable technologies as a facilitator, one of the telemedicine projects` objectives is to enhance access to health services for remote populations, particularly in tele-dentistry [7, 23, 24]. These technologies apply to support healthcare providers and patients in this area [10, 24, 27]. Another aspect that makes the use of affordable technologies an important consideration is that there are inequalities in the delivery of oral health services [67]. It appears that one of the useful means of reducing inequities is tele-dentistry applications with an emphasis on using technologies that increase user access to services.

Several studies have underlined the effective role of data quality in the diagnosis [9, 24], consultation [5, 24], and prioritization of patients in emergency conditions [19, 25] and documentation [5]. In addition, quality data includes features such as accuracy, timeliness, completeness, relevance, consistency, reliability and validity, and the presentation of data [68, 69]. This information is critical in supporting the diagnosis, treatment and measurement of the quality of patient care and improving and facilitating reimbursement [70].

Regarding the digital skills of patients and healthcare providers, studies have shown that the more skilled people use the technology, the more they will want to use it [11]. Communication skills, accurate documentation, and clinical experience are also considered as effective factors to optimize patient visit times, provide reliable diagnostics, and implement tele-consultation services in particular [5, 7, 11, 25].

### Barriers

The results showed that the barriers of tele-dentistry services were divided into six groups. Of these groups, more studies focused on technical and individual barriers.

In terms of technical barriers, most of the studies have concentrated on bandwidth problems and photo quality problems. Bandwidth problems have been raised in remote and rural areas, especially for oral cancer screening services that focus on underserved populations [1, 23, 24]. In tele-dentistry, image quality is an important factor in the correct diagnosis of oral disease [24, 26]. In this regard, to improve the quality of images, facilitators such as educating the patient for photography, providing guidelines for oral photography and using appropriate tools for photography were used [11, 39].

As for individual barriers, such as behavioral barriers and Lack of experience and skill has been addressed through some studies. For example, the lack of digital literacy is followed by the difficulty of using tele-dentistry services. So, it seems that education improvement as well as the use of user-friendly and convenient technologies is one way to mitigate that problem. Also, the difficulty of taking pictures of oral cavity in good light using a mobile phone led to a decrease in their willingness to use these technologies [11, 21]. Therefore, one of the ways to increase the patient's interest in tele-dentistry is to provide educational videos for taking photographs [11]. In addition, human errors and low-quality information are classified as personal and technical barriers, respectively. It appears that the use of artificial intelligence (AI) to adjust these factors could be an appropriate solution. In fact, AI provides enhanced remote screening, diagnosis, record keeping, triage, and monitoring of dental patients with smart tools [71]. AI can help dentists make critical and time-critical decisions. It can eliminate the human element of error in decision-making, providing a consistently high quality of healthcare [72].

### Participants` view

The results show that the perceived usefulness of tele-dentistry services in oral medicine was classified by users into 14 categories. Most of the studies have been conducted in the field of access to oral services. A study showed that for oral mucosal lesions, access to services has been improved in a timely and safe manner, while providing the opportunity to assess the patient's condition for pain relief, bleeding, and other postoperative complications using tele-dentistry [51]. This is also supported by another study, which suggests that tele-dentistry could improve access to services for people who are receiving cancer treatment. In this way, the side effects of patients can be followed by oral specialists using tele-dentistry [18]. As for continuity of clinical care, the results of a study demonstrated that patients were happy to continue their clinical care in unusual circumstances [5].

In terms of reasons for tele-dentistry services` dissatisfaction, lack of physical examination of the patient by doctors [10, 11, 20] is one of the problems that has received greater attention than others and has been raised by several studies. In a study which examined the patient's point of view and experience, the lack of physical examination was one of the barriers in the tele-dentistry, this case is not as serious in patients as in cases such as the difficulty of oral photography and the use of computer services [11]. In another study, it was reported that limiting physical exams was one of the problems in the investigation of patient experience, nevertheless, they had positive views on tele-dentistry [35]. Also, one study indicated that one of the greatest disadvantages of remote dentistry is the lack of physical examination [53]. This seems to have been compensated this problem with the use of high-quality photos. However, several studies have shown that face-to-face examination are the gold standard.

Studies have indicated the application of tele-dentistry dentistry in the COVID-19 crisis. In several studies tele-dentistry as an approach for facilitating access to oral medicine services during the COVID-19 pandemic for people with oral medicine conditions, which is in line with infection control protocols [22, 41, 62]. It is also important to follow up and provide advice to patients with oral medicine conditions, and some studies have indicated that this was one of the benefits of tele-dentistry during COVID-19 [12, 46]. In addition, one of the major issues addressed during the covid-19 and pandemic quarantine was the lack of unnecessary hospitalization, which was important in both directions to prevent the spread of the virus and to account for the high workload associated with COVID-19. In fact, tele-dentistry services have reduced unnecessary hospital referrals and saved resources by providing services for those with oral medicine problems [17, 63].

The review of selected studies has revealed several limitations that researchers can consider in their future research. The small sample size was suggested by numerous studies [3, 8, 15, 25, 40, 61, 62, 65, 73], Diagnostic error is also another limitation raised by studies, the reasons for which can be choosing histopathology as the standard gold [24], as well as the lack of oral examination due to the selection of new people [39]. One way to deal with this issue is to use tele-dentistry for patients` follow-up who have been examined on a face-to-face visit [39]. Also, other limitation including not comparing the provisional diagnosis of photography with the provisional diagnosis at in-person appointment for same patient [25], Possibility in remembering of diagnosis during clinical oral examination when reviewing the images taken using the mobile phone after weeks of washing out period [26], Limitations in diagnosis relying solely on visual examination [25], and Influencing of spectrum of lesions on the concordance values [26].

There are several suggestions for researcher, and everyone wants to develop the tele-dentistry services in the field of oral medicine:

• Focus on the quality of information shared (e.g., image, photo, document, video) in terms of accuracy, timeliness, comprehensiveness, and legibility.
• Using user-friendly and affordable technology for applying tele-dentistry services
• Development of a training program to improve users' level of digital literacy.
• Providing guidelines for oral cavity photography to improve quality of photos and accuracy of remote diagnosis, which reduce the problems of lack of physical examination, as one of the disadvantages of remote dentistry.
• Use artificial intelligence in teledentistry to reduce human error and improve the quality-of-care delivery at a distance.

## Limitation

This scoping review was limited to those articles which were published in English. As one of the main implications of the scoping reviews is in shedding the light for health policymakers’ future decisions, the results should be tailored according to the local context for the oral health policymakers and oral healthcare providers as the end users.

## Conclusion

In general, the results show that tele-consultation and tele-diagnosis services are among the top of tele-dentistry services that have been used in the field of oral medicine while new areas of applying tele-triage, remote screening, tele-visit, and telemonitoring services have been emerged during COVID-19 pandemic. The present framework also indicates that in the process of using tele-dentistry services in oral medicine, a category of information, clinical decision, skill, human resource, technical, administrative, financial, and training and education facilitators should be considered and at the same time, methodological limitations, individual, environmental, organizational, regulation, clinical, and technical barriers should be managed. Users` satisfaction and perceived usefulness of tele-dentistry as outcome can be increased considering the system`s feedback and applying facilitator incentives as well as decreasing the barriers.

## Supplementary Information

**Additional file 1.**

**Additional file 2.**

## Data Availability

All data generated or analyzed during this study are included in this published article and its supplementary information files (Appendix 1).

## Abbreviations

ICT: Information and Communications Technology
PCC: Population, Concept, Context
IPO: Input- Process-Output
