# Supplementary material for: Tele-dentistry, its trends, scope, and future framework in oral medicine; a scoping review during January 1999 to December 2021
Source: Arch Public Health. 2023 Jun 14;81:104. doi: 10.1186/s13690-023-01128-w (PMC10265557; doi:10.1186/s13690-023-01128-w)
Supplement: Supplementary file 1 — Additional file 1. [file 13690_2023_1128_MOESM1_ESM.docx]

**Appendix1:**

*Table 1- The search strategy of the scoping review*

| PUBMED | ("Teledentistry"[Title/Abstract] OR "Tele dental health"[Title/Abstract] OR "Tele oral health"[Title/Abstract] OR "E-oral health"[Title/Abstract] OR "Remote dentistry"[Title/Abstract] OR "Teledentistry platform"[Title/Abstract] OR "Telehealth platform "[Title/Abstract] OR "Teledentistry Model"[Title/Abstract] OR "Tele-oral medicine"[Title/Abstract]) AND ("Oral Medicine"[Title/Abstract] OR " Stomatology"[Title/Abstract] OR " Medicine Oral "[Title/Abstract] OR "diagnosis "[Title/Abstract] OR "oral lesions"[Title/Abstract] OR "lesion of oral"[Title/Abstract] OR " Oral Manifestations "OR "Oral diseases "[Title/Abstract] OR " mouth diseases"[Title/Abstract] OR "Orofacial disorders "[Title/Abstract] OR " orofacial pain "[Title/Abstract] OR "oral mucosal diseases"[Title/Abstract] OR " salivary gland disease"[Title/Abstract] OR " oral cancer "[Title/Abstract]) |
| --- | --- |
| SCOPUS | ( TITLE-ABS-KEY ( "Teledentistry" OR "Tele dental health" OR "Tele oral health" OR "E-oral health" OR "Remote dentistry" OR " ‘Teledentistry platform" OR "Telehealth platform " OR "Teledentistry Model" OR "Tele-oral medicine" ) AND TITLE-ABS-KEY ( "Oral Medicine" OR " Stomatology " OR " Medicine Oral " OR "diagnosis " OR "oral lesions" OR "lesion of oral" OR " Oral Manifestations" OR "Oral diseases " OR "mouth diseases" OR "Orofacial disorders" OR "orofacial pain " OR "oral mucosal diseases" OR "salivary gland disease" OR "oral cancer " ) ) |
| ISI Web of Science | (TS=("Teledentistry" OR "Tele dental health" OR "Tele oral health" OR "E-oral health" OR “Remote dentistry" OR " ‘Teledentistry platform" OR "Telehealth platform " OR "Teledentistry Model” OR “Tele-oral medicine” )) AND TS=("Oral Medicine" OR " Stomatology "OR " Medicine Oral "OR " diagnosis " OR " oral lesions " OR "lesion of oral" OR " Oral Manifestations "OR "Oral diseases " OR " mouth diseases" OR" “Orofacial disorders " OR " orofacial pain " OR "oral mucosal diseases" OR " salivary gland disease" OR " oral cancer ") |
| PROQUEST | ("Teledentistry" OR "Tele dental health" OR "Tele oral health" OR "E-oral health" OR "Remote dentistry" OR " ‘Teledentistry platform" OR "Telehealth platform " OR "Teledentistry Model" OR "Tele-oral medicine") AND ("Oral Medicine" OR " Stomatology " OR " Medicine Oral " OR " diagnosis " OR " oral lesions " OR "lesion of oral" OR " Oral Manifestations " OR "Oral diseases " OR " mouth diseases" OR " Orofacial disorders " OR " orofacial pain " OR "oral mucosal diseases" OR " salivary gland disease" OR " oral cancer ") |
| Search strategy | #1 :  “Teledentistry" OR "Tele dental health" OR "Tele oral health" OR "E-oral health" OR “Remote dentistry" OR " ‘Teledentistry platform" OR "Telehealth platform " OR "Teledentistry Model” OR “Tele-oral medicine”  #2:  "Oral Medicine" OR " Stomatology "OR " Medicine Oral "OR " diagnosis " OR " oral lesions " OR "lesion of oral" OR " Oral Manifestations "OR "Oral diseases " OR " mouth diseases" OR “Orofacial disorders " OR " orofacial pain " OR "oral mucosal diseases" OR " salivary gland disease" OR " oral cancer "  #3: #1 AND #2 |
| Mesh Term | -oral medicine, Stomatology, medicine oral, mouth diseases, oral Manifestations  - orofacial Pain  -Salivary Gland Disease  - Oral Cancer |
| Limits | language: English, Time: *1999-01-01 – 2021-12-30.*  *Date of search: December2021* |

Table 2: Summary of the characteristics of included studies,1999-2021

|  | Author | country (year) | Aims | type of study | | population | Study setting | Sample  size | Methodology | Main findings and implications |
| --- | --- | --- | --- | --- | --- | --- | --- | --- | --- | --- |
| 1 | R. Amtha | Indonesia (2021) | investigate the level of satisfaction | observational cross-sectional study | | Oral medicine patients who used teledentistry services at the start of the COVID- 19 pandemic. | Oral Medicine Clinic Dental Hospital, Faculty of Dentistry Universitas Trisakti, Jakarta | n=31 | - Fill out the questionnaire by patients who used teledentistry services at the beginning of the COVID-19 pandemic - **satisfaction scores:**   very dissatisfied; not  satisfied; moderate; satisfied and very satisfied   - using the Rasch model to validate the questionnaire - using the analysis factor to determine the component that contributes to satisfaction | **Level of satisfaction:**  100 %of the subjects were in the satisfied and very satisfied categories  **contributing factors:**  the components of comfort and convenience and communication between patients and doctors. |
| 2 | G. Tenore | Italy  (2021) | evaluate patients’ perception and acceptance of a tele(oral)medicine program in the COVID-19 pandemic. | cross-sectional pilot survey | | Patients treated at the MoMax (Oral Medicine and Maxillofacial) | MoMax ambulatory at the Department of Oral Sciences and Maxillofacial Surgery at “Sapienza” University of Rome, Polyclinic Umberto | n=84 | - conducting a telephone survey on 84 patients - recording of the demographic characteristics of the patients - complete the 24 question-survey - Questionnaire feature:   24 multiple-choice questions divided into four different domains: sociodemographic variables; factors that may influence the patient’s experience within the MoMax ambulatory; skills in the use of technology a; and interest in tele(oral)medicine services. | - **Significative statistical associations**: - between patients’ interest in a tele(oral)medicine service and computer skills, conditions influencing the experience of the conventional visit, and the   difficulty in taking intraoral photos.   - the tele(oral)medicine program help to meet the expectations and needs of our patients |
| 3 | A. Villa | USA  (2021) | Introduce Tele(oral)medicine as a new approach in the COVID-19 pandemic. | letter to the editor | | Patients with oral medicine conditions | Department of Orofacial Sciences,  University of California San Francisco | NA | Describe a practical application of Tele(oral)medicine in covid-19 pandemic | - Telemedicine clinical exams lack tactile assessment - telemedicine it is a good system for continuity of care, the ability to prioritize patient medical needs, and the potential to reduce patient anxiety related to delays in scheduling their office visit. |
| 4 | Maha Ali Al Mohaya | Saudi Arabia  (2021) | assess the use of telemedicine among oral medicine practitioners in Saudi Arabia during COVID-19 pandemic. | Cross-sectional survey study | | different professional levels of oral medicine involving consultants, specialists, and residents. | - | n=75 | - perform a self-administered supplement-based survey - **The supplement content:**   four main sections including demographic data, the current use of telemedicine, potential barriers and limitations of telemedicine in the specialty practice and participant’s perspective on future implications.   - measurement of the views on possible future implementation - using five-point Likert scale | **Reason for the use of telemedicine:**   - consultation, before COVID-19 outbreak (50.7%) - consultation, during (72%) COVID-19 outbreak, - communicating with patients 57.3% - 45% patients were satisfied (α = 0.82). - Poor knowledge and training in telemedicine technologies affect significantly the future implications of telemedicine in the practice of oral medicine(p<0.05). - application of telemedicine in oral medicine practice, especially for remote diagnosis, consultation, referral, training and education |
| 5 | B. B. Fonseca | Brazil  (2021) | Evaluate telediagnosis of oral lesions by using smartphone photography | cross-sectional, observational study | | Individuals with visible oral lesions composed a convenience sample. | Oral Medicine Clinic of the Federal University of Paraná. | N=113 | - Take photos of the lesions with smartphone camera. - Email photo along with clinical information to three evaluators. - formulating up to two diagnostic hypotheses for each case by evaluators. - comparing diagnostic hypotheses to the gold standard by means of percent agreement and kappa coefficient | - perfect agreement and diagnostic accuracy comparable to face-to-face diagnosis. Kappa coefficients   (k = 0.817–0.903)   - supporting the referral process from primary to secondary care in oral medicine |
| 6 | Vignesh Murthy | UK  (2021) | Evaluate patient experience of virtual consultations in the Oral Medicine department during the first wave of the COVID-19 pandemic. | Survey | | All patients attending virtual appointments in the Oral Medicine department at GSTT on the NHS Attend Anywhere virtual consultation platform and telephone consultations | Oral Medicine department at GSTT on the NHS | n=115 | - a validated survey was complete by 115 patients following their virtual (telephone or video) consultation - Data were synthesized and electronically analyzed - Qualitative data were thematically analyzed | Over 82% had good or very good experience 69% preferred a virtual consultation for their next consultation. |
| 7 | I. D. A. F. Muniz, | Brazil  (2021) | concentrate the importance of teledentistry on diagnosis and patient management. | Case report | | An old female patient with oral mucosa garlic burns. | - | n=1 | During the teleconsultation, the patient stuck out her tongue in front of the camera, took pictures and forwarded them directly via the WhatsApp application | Importance of tele dentistry during a pandemic for oral diagnosis and treatment |
| 8 | Maret, D., | France  (2021) | Providing a technical note to help clinician, which is for advising patients about photography of oral cavity | Technical note | | - | - | NA | NA | provide a technical note to take photo of oral cavity |
| 9 | Sirikanlaya Vetchaporn | Thailand  (2021) | Evaluate the validity and reliability of the intraoral camera with the combination method of autofluorescence and LED white light used for OPMDs and oral cancer screening in teledentistry. | Feasibility  study | | Patients who had signed the informed consent documents were recruited from the Oral Biology and Oral Diagnosis Clinic, Faculty of Dentistry, Chiang Mai University, from December 2020 to March 2021 | Patients with oral lesion condition | n=34 | - There were 3 parameters of the data to be analyzed including (1) category of lesion, (2) dysplasticity of lesion, and (3) image score. - percent agreement statistic was used for evaluation of diagnostic concordance as reliability. - Oral medicine specialist was accessed for all images by reviewing online via “Line Application” using the same smartphone. | - the agreement between clinical direct examination and images reviewing from the devices via teledentistry for determining the category of lesion on initial diagnosis. Kappa coefficients (k=0.794) - intraoral camera with fluorescent aids for the OPMDs screening can be utilized for screening via teledentistry |
| 10 | Praveen Birur N | India  (2021) | Assess the ability of CHWs to identify oral mucosal lesions using mobile technology and determine agreement on the detection of oral lesions between CHWs using mHealth and onsite oral medicine specialists Vs remote oral medicine specialists. | A cross-sectional analytical study | | Participants were screened/due to high risk of tobacco usage reported | workplace setting.in a pipeline factory | N=3445 | - The participants were screened by two CHWs, and subsequently assessment by an oral medicine specialist. - A mobile phone‑based questionnaire that included the risk assessment was distributed among participants. - histopathology was considered as gold standard. - Sensitivity, specificity, positive and negative predictive values were calculated. Inter‑rater agreement was analyzed with Cohen’s kappa coefficient (κ) test - the diagnostic ability of CHWs, onsite specialist, and remote specialist was illustrated using receiver operating characteristic curve | - perfect agreement between the CHW and the onsite specialist.   κ =0.92   - substantial agreement between CHW and remote specialist. k= 0.62. - the values of CHWs in the identification of oral lesion:   **sensitivity= 84.7**  **specificity=97.6**  **positive predictive=84.8**  **negative predictive=97.7%** |
| 11 | Alona Emodi-Perlman Alona | Israel  (2021) | Review of the initial existing literature on TMD, bruxism and orofacial pain during the COVID-19 pandemic – signs and symptoms, triage, diagnosis, and management during times of psychological tension, immediate health hazards and social isolation | | Review | - | - | NA | - performing a literature search about 10 months after the declaration of the pandemic - finding a few studies dealing with TMD and bruxism during COVID-19. | - describe applications of teledentistry   - remote modes for the triage, diagnosis  and treatment of chronic orofacial pain patients. |

Continue Table 2

|  | Author | country (year) | Aims | type of study | population | Study setting | Sample  size | Methodology | Main findings and implications |
| --- | --- | --- | --- | --- | --- | --- | --- | --- | --- |
| 12 | Krishna S. Kumar, | India (2021) | Assess the guidelines of safe Oral Medicine practice in the COVID-19 pandemic situation including the efficient management of emergencies, a multidisciplinary approach for the management of oral mucosal lesions, discuss on the reported oral manifestations in patients with COVID-19 infection | scoping review | - | - | NA | - literature search was done using Medline/PubMed database as a search engine with standard - There was no restriction on the type of study design | utilization of teledentistry is an important contributor to the safe practice of oral medicine during the COVID-19 pandemic. |
| 13 | Alba Pérez González | Spain  (2021) | Review applications of teleconsultation in the different fields of dentistry, and analyses dentists’ perceptions, patient acceptance, the efficiency and cost effectiveness, as well as the most important ethical and legal aspects in this field. | book  chapter | - | - | NA | - | - review the different types of teleconsultations and the importance in the current pandemic, continuing with the applications of teleconsultation in the different fields of dentistry - analyses dentists’ perceptions, patient acceptance, the pros and cons of using teleconsultation - describe the most important ethical and legal aspects in teledentistry |
| 14 | Shantanu Deshpande | India  (2021) | Discusses teledentistry and its applications in general and specialty dental practice amidst the COVID-19 lockdown. | Narrative review | - | - | NA | - | Application of teledentistry in Oral Medicine to manage emergency conditions, consultation and t treatment plan during the lockdown period |
| 15 | Miao Xian Zhou | USA  (2021) | Report a case with non-Hodgkin’s lymphoma manifesting and the role teledentistry the management of diseases | Case report | A 67-year-old female with Graves’ disease and non-Hodgkin’s lymphoma | Department of Dental Specialties | n=1 | Uploading photo on the portal by patient and a video visit via Zoom platform to discuss her concerns | - Useful in covid-19 pandemic - useful for screening and visiting people with mobility impairments in remote areas - for the early detection and diagnosis of oral cancer |
| 16 | Na Lv | China  (2021) | Discuss on the management of oral medicine patients during COVID-19 emergency and concentrating on remote assistance devices and classification of emergencies | Review | - | - | NA | using digital technologies, such as video conferencing with Zoom, Google Meeting or WhatsApp during the lockdown period. | Digital technologies, useful and efficient tools that oral medicine practitioners could consider for patient triage, managing emergencies, reassure, and remote patient monitoring. |
| 17 | Alessandro Villa | USA  (2021) | assess patient and provider's satisfaction, experiences, acceptance and engagement in using tele(oral)medicine during the COVID-19 pandemic | cross-sectional study | Patients and oral medicine provider | three oral medicine centers in the United States that started tele(oral)medicine since the beginning of the COVID-19 pandemic | Patients: n=100  Providers:  n=9  visits: n=108 | - patients and oral medicine specialists responded to the survey for a total of 108 visits. - the survey queried on overall satisfaction with the video appointments via Zoom, comfort level with the video technology (quality of the video and sound) and willingness to use telemedicine in the future | - Patients and providers were well satisfied with tele- oral medicine - Tele -oral medicine has proven to be a convenient and effective technology for providing health care to people who have limited access to care from oral medicine specialists. |
| 18 | Jitendra Chawla | India  (2021) | Describe the ability of Tele-Oral medicine to detect oral cancer early | letter to the editor | - | - | NA |  | The usefulness of using of telemedicine in the continued early diagnosis and prevention of oral and pharyngeal cancer |
| 19 | Delphine Maret | France  (2021) | report a case and the role of telemedicine contributing to an incidental finding of a premalignant lesion | Letter to the editor/case study | An 87-year-old man patient with actinic cheilitis localized on the lower lip | - | n=1 | - Taking pictures of the patient's mouth with a mobile phone - Send photos to the specialist | - usefulness of teledentistry in emergency situations - the photographs can easily show the patient’s mouth in sufficient quality - having a good view of all the anatomical parts that are within the competence of a dental surgeon is important |
| 20 | I. Lin, | Canada  (2021) | 1. To describe how IO photography can be used in teletriage of high-risk oral lesions and continued follow-up of low-grade dysplasia. 2. To establish recommendations for obtaining good quality photos in the clinical and home setting. | Scientific Research Report | - | In British Columbia (BC), Canada | NA | NA | Recommendation methods to photography in high quality way |
| 21 | Anna Luíza Damaceno Araújo | Brazil  (2021) | proposing validation of remote WSI(whole slide images) assessment and reporting for the diagnosis of oral and maxillofacial pathologies | Feasibility study | glass slides from patients | - | glass slides:  n= 162  patient:  n=109 | - A total of 162 slides from 109 patients included in this validation over a period of 5 weeks were evaluated by all the pathologists in the two analyzes (digital and conventional).   washout period: 1 month   - measurement of the intraobserver agreement between Digital and conventional method | - the perfect agreement between the digital method (DM) and conventional method (CM) :κ= 0.85 to 0.98, with 95% CI, ranging from 0.81 to 1). - use of online meeting tools are valuable resource during the Covid-19 pandemic and the post-pandemic. - ensuring the feasibility of the remote histopathology this study |

Continue Table 2

|  | Author | country (year) | Aims | type of study | population | Study setting | Sample  size | Methodology | Main findings and implications |
| --- | --- | --- | --- | --- | --- | --- | --- | --- | --- |
| 22 | Camille Inquimbert | France (2021) | to determine if teledentistry consultations using fluorescence are of the same quality as regular consultations in the diagnosis of caries | Feasibility study | Patients who  had a scheduled  consultation with the dental center at the Montpellier  University Hospital (France) or the Kyushu Dental  University Hospital (Japan). | the dental care center at the Montpellier University Hospital (France)  and in the center at Kyushu Dental University Hospital (Japan). | Patient  N= 200 | - Patients were seen in consultation in two dental care center - The study protocol was included three step: - the regular consultation - the recording of videos with the Soprocare camera by two deferent dentists - the remote consultation, the videos were all rendered anonymous before being analyzed blindly by a third dentist in six months - measurement of diagnostic performances of the remote consultation in the detection of caries. | - diagnostic performance of teledentistry consultations: acceptable diagnostic performance with regard to the detection of dental caries. - early diagnosis of carious lesions with optimal efficiency by the Soprocare camera |
| 23 | N. Dar-Odeh | Jordan  (2020) | analyze Jordanian dentists’ inquiries on oral infections and antimicrobial prescribing using dental professional WhatsApp groups during covid-19 lockdown period | cross-sectional study | Three professional WhatsApp groups of Jordanian dentists | - | n=43  queries posted | - Three professional WhatsApp groups of Jordanian dentists were reviewed and analyzed for inquiries. - All queries regarding oral infections and antimicrobial prescribing were - extracted and analyzed. | Use of WhatsApp group for consultations on oral diseases, prescription and dental care for medically compromised patients. |
| 24 | G. M. Gillligan, | Argentina (2020) | Report a case reflecting a suitable use of telemedicine in Oral Medicine | letter to the editor | A case with chief complaint of spontaneous gingival bleeding, with no previous medical conditions | Oral Medicine Department, Facultad de  Odontologia, Universidad Nacional de Córdoba | n=1 | A general dental practitioner, working in a small  Patagonian village referred specialist a case using Instagram and WhatsApp. | - future research efforts should address Telemedicine as a diagnostic method - take into account relevant questions such as the quality of the photographic record, legal and remunerative aspects. |
| 25 | J.H. Macken | UK  (2020) | describe experiences and an approach to use remote telephone clinics in oral medicine | Short communication | - | Remote telemedicine clinics in Oral Medicine (OM) at the London Behc¸et’s Centre of Barts Health NHS Trust | NA | NA | - The advantages of teleconsultation including Ongoing clinical care, Connection with the service, Patient empowerment, self-monitoring and risk-factor modification and Learning opportunities for trainees - important factor in teledentistry: communication skills, clinical reasoning, differential diagnosis construction, documentation |
| 26 | Marcio AjudarteLopes, | Brazil  (2020) | Introduce virtual assistance to patients with cancer and to health care professionals | letter to the editor | Patients with Oral cancer | - | NA | - | application of teledentistry for patient patients with cancer and to health care professionals, particularly dentists and prioritize higher-risk cases |
| 27 | Eleni A. Georgakopoulou | Greece  (2020) | describe the role of digitally aided telemedicine during the SARS-CoV-2 pandemic to screen oral medicine emergencies | letter to the editor | Patients with oral medicine emergencies | - | n=16 | - using available software resources that allow users - Patients submitted a variety of test results, medical history, and GDPR approvals for information processing. - making electronic prescription. - to share media like viber, email , Messenger | To minimize unnecessary visits during this global pandemic, digitally assisted telemedicine can be an effective way to monitor oral medical emergencies. |
| 28 | Maria Inês MEURER | Brazil  (2020) | describe tele(oral)medicine model in Brazil on COVID-19 pandemic | letter to the editor | population of State of Santa Catarina | The State of Santa Catarina is located in the South of Brazil | NA | Describe a tele(oral)medicine model | The telemedicine (oral) module has been implemented in the STT/SC web system and supports the security criteria required for the transmission of sensitive patient data via the network, in accordance with Brazilian regulations such as confidentiality/encryption, authenticity, integrity, irrefutability. and timestamp |
| 29 | Risha Sanghvi | UK  (2020) | introduce alternative assessment and communication methods for management of patients with oral  conditions | cross-sectional study | Patients on the waiting list for the joint oral medicine pediatric dental clinic | Guy’s Hospital and St Thomas’ Hospital. | n=57 | - patients on the waiting list for the joint oral medicine pediatric dental clinic were prioritized on including criteria - A telephone consultation method was chosen to assess and follow up patients and a parental satisfaction questionnaire was conducted following telephone consult | - 100% of patients preferred telephone consultations to face-to-face visits during COVID-19 - 74% preferred a video consultation over a phone consultation - telephone consults to be a valuable alternative to face-to-face consults in the management of patients with chronic oral mucosal diseases. |
| 30 | Fabio A. Alves | Brazil, Canada, and the United States  (2020) | Discuss the clinical workflows of cancer center-based Oral Medicine practices in covid-19 pandemic | letter to the editor | - | cancer center-based Oral Medicine practices in Brazil, Canada (Northeast Cancer Centre, Health Sciences North, Sudbury), and the United States (Dana-Farber/Brigham and Women's Cancer Center, Boston | NA | - | - using teledentistry to facilitate patient–professional communication and has been extensively and effectively incorporated into oncology care. - facilitate the provision of oral medicine care, support teleconsultation and the effective triage by Virtual visits |
| 31 | N. Haron, R | Malaysia  (2020) | evaluate the feasibility of using Mobile Mouth Screening Anywhere (MeMoSA) to facilitate early detection of OC. | feasibility study | individuals who are of age >18 years old. | a village in Peninsular Malaysia where the nearest tertiary referral center was 42 km away. | n=48 | - developing a mobile phone app named MeMoSA - evaluation the feasibility of integrating this for documentation of oral lesions, and communication between dentists and specialists for management decisions - Determine the experience of dentists and specialists using MeMoSA through qualitative questionnaires. | - from dentists’ point of view, MeMoSA could facilitate the early detection of oral cancer. - from dentists’ point of view, MeMoSA could assist in the identification of oral mucosal lesions via direct communication with specialists and continuous learning in the recognition of high-risk lesions using MeMoSA stratified cases and streamlined referral of patients |
| 32 | N. N. Perdoncini | USA  (2020) | evaluate the feasibility and accuracy and patient satisfaction of real-time teleconsultation | feasibility study | Patients referred for specialized care owing to oral lesions | the oral medicine clinic of the Federal University of Paraná | n=33 | - Take photos of the lesions with smartphone camera - sent photos via WhatsApp application to an oral medicine specialist, with whom a video call was initiated on WhatsApp and formulating up diagnostic hypotheses for each case . - a second specialist, blinded to the first evaluation, assessed the oral lesion in person and defined a diagnosis, which was considered as the reference standard. | - the concordance between the telediagnosis and the reference standard (k = 0.922).   -Men were significantly more satisfied with dental services than women  - high-quality internet connection has been important role for greater user satisfaction |
| 33 | M. Roxo-Gonçalves | Brazil  (2020) | usability evaluation of the EstomatoNet Platform and identify user perceptions | observational cross‐sectional study | Dentists | Brazilian Unified Health System | dentist:  n=16 | - To assess usability, participants have requested telediagnosis support for a fictional case provided by the research team. - uploading the information and sending the request, users were asked to “think out loud,” expressing their perceptions. - observing the session by an examiner with remote access to the user’s screen (via Skype). - completing of the System Usability Scale (SyUS) After the simulation | - The EstomatoNet Platform has satisfactory usability. - Some key issues with information fields need to be resolved to improve the tool. |

Continue Table 2

|  | Author | country (year) | Aims | type of study | population | Study setting | Sample  size | Methodology | Main findings and implications |
| --- | --- | --- | --- | --- | --- | --- | --- | --- | --- |
| 34 | Pierre Binaisse | France  (2020) | evaluate the frequency, characteristics and management of dental emergencies at sea in France. | descriptive study | records of patients who were assisted by the French maritime TeleMedical Assistance Service (TMAS) from 2012 to 2016 | emergency medicine  centre of the University Hospital of Toulouse | medical files  n=9122 | - Ranking of data in different categories: socio-demographic data, diagnosis, prescription, and monitoring or treatment prescribed. | - the total number of medical files recorded by TMAS: 9122 - the number of medical records concerned oral diseases= 135 - Management of dental emergencies among TMAS, the different prescriptions were ranked according to the main diagnosis |
| 35 | Amerigo Giudice | Italy  (2020) | description of the advantages of telemedicine (TM) in dental practice during the current national emergency condition due to the Covid-19 dissemination | A Descriptive  Pilot Study | patients with urgent pathologies (U group) and  patients in follow-up (F group) | At Department of Oral Surgery and Pathology—Magna Graecia University of Catanzaro, regional  reference center for Covid-19 | Patient  N=57  Photos  N=418 | - running remote consultations using WhatsApp Messenger to send photos by both groups | - Advantages of Telemedicine :   monitoring of all patients, reducing costs and limiting human contact, decreasing the risk of Covid-19 dissemination. |
| 36 | Praveen Birur N | India  (2019) | describe a Novel Mobile-Health Approach to Early Diagnosis of Oral Cancer | cohort | high-risk individuals in door-to-door and workplace settings | two villages in Karnataka | n=42754 | - screening of high-risk individuals in door-to-door and workplace settings - capture of images of suspected lesions. - interpret uploaded data and send specialist recommendation from a remote location. - recommendation was intimated to FHPs who arranged for further action. | - Electronic data collection facilitates effective monitoring. - The program was very cost-effective with screening completed under $1 per person. - challenges: securing participation of populace in screening, and in compliance for biopsies. Technological hurdles included poor connectivity in some areas |
| 37 | Kalaiselvi Vinayagamoorthy | India  (2018) | assess the feasibility of using photo messaging as a tool in the preventive screening for oral PMDs | observational cross‐sectional study | participants of oral screening programs | Primary care setting in Udupi District, Karnataka, South India | Patients:  n=131  image:n=655 | - taking photo of lesion of patient by phone and send with WhatsApp - The reliability was tested at two levels. At the general level, lesions were categorized as normal and abnormal. At the specific level, the reliability of an exact diagnostic match of lesions between the examinations was considered. | When lesions were categorized as normal and abnormal, the agreement between the diagnoses, based on photo messaging and clinical oral examination  **kappa coefficients**:  Examiners 1 : (k= 0.68 )  Examiners 2: k=(0.67)  **specificity:**  Examiners 1= 72%  Examiners 2= 64%,  **Sensitivity:**  Examiners 1= 98.5%  Examiners 2= 99.04%,  When the agreement between photo messaging and clinical oral examination for an exact diagnostic match  **kappa coefficients:**  Examiners 1 : (k= 0.59)  Examiners 2: k=(0.55)  **specificity:**  Examiners 1= 64%  Examiners 2= 52%,  **Sensitivity:**  Examiners 1= 98.1%  Examiners 2= 98.7%, |
| 38 | Eduardo K. Kohara | Brazil  (2018) | compare the performance of two different models of smartphone and a conventional camera with that of direct clinical examination in detecting caries  lesions at different stages of progression in deciduous molars | feasibility study | Fifteen children aged 3 to 6 years | Clinic of the Department of Pediatric Dentistry, School of Dentistry, University of São Paulo. A | Children  N=15 | - The photographic equipment: - iPhone , Nexus 4, and a conventional macro camera - all of the photographic images were taken using the photographic equipment - In both groups, two examiners, blinded to the photographic equipment used, assessed the images independently on a computer screen, and classified them according to ICDAS. - reference standard: Consensus of opinions two experienced examiners | - the highest values of correct answers: for sound and extensive caries lesions in both laboratory and clinical settings. - the lowest value of correct answers: for initial and moderate lesions in the clinical evaluation, irrespective of the camera devices used. - photographic diagnosis using smartphone images is feasible and accurate for distinguishing sound tooth surfaces |
| 39 | N. Haron, R | Malaysia  (2017) | measure the concordance in clinical diagnosis between COE and images taken with the mobile phone | feasibility study | individuals with a range of oral potentially malignant disorders (OPMD) and normal oral mucosa | Faculty of Dentistry, University of Malaya | Patients:n=16  OMS: n=2  dentist: n=2 | - photography of Five areas of the oral cavity by three dentists using mobile phone cameras with 5 MP-13 MP resolutions. - clinical oral examination (COE) of patients on the same day by two oral medicine specialists - review of the photos 3 weeks later using the phone by oral medicine specialists - examine the concordance between the two by Kappa statistics. - measure the sensitivity and specificity of the clinical diagnosis using telephone images - completing of pre- and post-program questionnaires by both the dentists and the OMS to determine the feasibility of integrating teledentistry in their clinical practice | - For determining the presence of lesion, category of lesion (OPMD or not), and making referral decision:   **Kappa coefficients:** moderate to strong (0.64–1.00),  **overall sensitivity:** more than 70%  o**verall specificity:** 100%.  **The false negative rate** decreased as the camera resolution increased   - teledentistry can be used for communication between primary care and OMS - teledentistry can be easily integrated into the clinical context for patient management |
| 40 | VC Carrard | Brazil  (2017) | introduce WhatsApp as telemedicine platform for facilitating remote oral medicine consultation and improving clinical examinations | letter to the editor | - | - | NA | - NA | Describe a practical application of tele dentistry |
| 41 | M. Roxo-Gonçalves | Brazil (2017) | evaluate the diagnostic skills of primary healthcare professionals on oral cancer | analytical cross-sectional study | primary healthcare professionals | International Agency of Research in Cancer (IARC) | n=47 | - 32 dentists and 15 non-dentists enrolled in a 24-h course on oral medicine delivered through an e-learning platform. - the use of 333 clinical images obtained from the digital manual for the early diagnosis of oral to evaluate the diagnostic skills of participants. - classification of each lesion as benign, potentially malignant, or malignant - gold standard: the specialist opinion - Comparison between the diagnosis skills of dentist and non-dentist | **Sensitivity:**  Dentist: 63.7 – 15.8  Non-dentist: 68.8 – 11.1  **Specificity:**  Dentists: 70.0% – 16.6%  Non-dentist: 39.3–20.6  specialists: 95.5% – 3.1%   - both dentists and non-dentists have a fairly good capacity for discriminating the nature of oral lesions. |
| 42 | Alain Queyroux | France  (2017) | Evaluation of the accuracy of teledentistry for the diagnosis of dental pathologies, evaluation of the Denture rehabilitation status and evaluation of chewing ability of elderly people living in nursing homes. | feasibility study | Nursing home residents with oral or dental complaints | Eight nursing homes in France and Germany. | Patient  N=235 | - The patients were examined twice:  1. dentist established a diagnosis by reviewing a video recorded in the nursing home and accessed remotely. 2. in a maximum of 7 days, patients were examined conventionally by the same dentist  - comparing the diagnoses established via the video recording and in the face-to face setting | - The sensitivity of teledentistry for diagnosing dental pathology:   93.8% (95% confidence interval [CI] 90.7-96.9),   - the specificity of teledentistry for diagnosing dental pathology :   94.2% (95% CI 91.2-97.2).   - Teledentistry has shown excellent accuracy for diagnosing dental pathology in older adults living in nursing homes - teledentistry use may allow more regular checkups to be carried out by dental professionals |
| 43 | Mohamed Estai | Australia (2017) | to evaluate users’ acceptance of a teledentistry model utilizing a smartphone camera used for  dental caries screening and to identify a number of areas for improvement of the system | survey | Participants (users): Graders (dental practitioners)  Teledental assistants (smartphone users) | Australian  E-Health Research Centre (AEHRC) | User: 22  Smartphone user: 17 | - development a telemedicine platform ‘‘Remote-I’’ to assist in the screening of oral diseases - sending 485 images (five images per case) from the Android app to the server. - image assessment by dental practitioners (graders) - reporting the diagnosis after assessment by dental practitioners (graders) - sending a user acceptance survey to the graders and smartphone users following completion of the screening program | - user satisfaction: Users satisfied with the proposed teledentistry model. - time to read image by graders: less than 15 min for the majority of graders - time to complete the dental photography using the Android app: 5-10 min by phone user - factors that are essential for improving the current system:   optimization of smartphone camera features, the format of the server, and the orientation of images and using oral retractors during photography. |
| 44 | Mohamed Estai | Australia (2016) | To evaluate a cloud-based telemedicine application for screening for oral diseases. | feasibility study | six volunteers were enrolled in a trial | Australian  E-Health Research Centre (AEHRC) | N=6 | - Development a telemedicine system, based on a store-and-forward method, - development an android application to facilitate entering demographic details and capturing oral photos. - enrolling six volunteers in a trial to obtain oral images using smartphone cameras. - Obtaining images of the participants' teeth by a trained dental assistant after an oral examination on site - uploading oral images directly from the smartphone to a cloud-based server via broadband network. - comparing the assessments of oral images by offsite dentists with those carried out via face-to-face oral examinations. | - Sensitivity teledental screening: 57% - specificity teledental screening: 100% - The inter-grader agreement estimated for two examination modalities:70% - The inter-grader agreement estimated for two teledental graders: 62% - the proposed system for screening of oral diseases can be implemented to provide a valid and reliable alternative to traditional oral screening. |
| 45 | M. Petruzzi | Italy  (2016) | describe use of the WhatsApp to share clinical oral medicine information | cross-sectional study | patient referrals to specialized oral pathology and medical centers | specialized oral pathology and medical centers in Southern Italy | clinical image:  339 of 96 case by 11 patients | Sending the Clinical images and related questions to the authors via WhatsApp by general dentists, doctors, dental hygienists and patients | - The most common question was related to diagnosis (56%). - The telemedicine impression agreed with the clinicopathologic assessment for 82% of cases. - supporting of communication about oral conditions among clinicians and patients by using WhatsApp |
| 46 | Vinicius Coelho Carrard | Brazil  (2016) | summarize the experience of the EstomatoNet, a telediagnosis program | exploratory cross-sectional study. | All clinical cases submitted by PHC dentists or physicians to EstomatoNet | municipalities in the state of Rio Grande do Sul | n=259 | - Healthcare providers submitted requests including clinical information and photographs of oral lesions using a cloud-based platform. - Oral medicine teleconsultants received data, passed on a diagnostic hypothesis, and made management recommendations - Tool for measurement the outcomes:   **Level of satisfaction and impact of EstomatoNet**: self-administered questionnaire to evaluate the service  **Quality of photos**: Photo evaluation with two examiners  **Time elapsed for the responses:** The mean time elapsed from the entire process | - **Level of satisfaction:** very satisfied (98%) - **Quality of photos:** Most photos (n=251, 96.9%) were considered adequate - **Time elapsed for the responses:**   81.9 ± 67.8h   - Using of telediagnosis for oral lesions is feasible - Telediagnosis can improve the quality of primary care by bridging the gap between primary and specialty care. |
| 47 | Martha Tesfalul | USA  (2016) | assess the system’s impact on the patients’ diagnoses and management plans | observational study | Records for adult patients receiving care via the mobile oral telemedicine system | Botswana | n=26 | - The diagnoses and management plans proposed by the dental officers referring cases were assessed for concordance with those put forward by the specialists. - The inferred resource utilization of the two management plans was also assessed for concordance. - Concordance was assessed as a categorical variable with values of Concordant, Discordant, and Unable to Determine | -high diagnosis concordance between dental officers and oral health specialists at 91.3% (21/23)  -significant management plan discordance at 64.0% (16/25),  -mobile telemedicine can optimize the use of insights and skills of specialists remotely in regions where they are scarce |
| 48 | Birur PN | India  (2015) | develop a mobile phone–based platform for risk stratification and evaluation of the mouth by remote oral cancer specialists | cohort | participants with a high prevalence of oral cancer risk habits | rural villages, Anakanur and Poshetahalli , of the hikkabalapur district in the suburbs of Bangalore City. | Cohort2: (opportunistic; n = 1,440)  Image:n=106  Cohort1:  targeted cohort :(n=2,000) | - The study population included a targeted cohort and an opportunistic cohort screened by FHW and dental professionals, respectively. - compare the screening efficacy in both groups, with specialist diagnosis considered the reference standard. - outcomes: lesion detection and capture of interpretable images of the oral cavity | **In targeted cohort:** among 51 of 81 (61%) interpretable images, 23 of 51 (45%) of the lesions were confirmed by specialists  **the opportunistic cohort:**  100% concordance with the specialists (106 of 106)   - mobile health–based approach, is a step toward a more effective oral cancer screening program |
| 49 | Cassius C. Torres-Pereira | Brazil  (2015) | summarize the telehealth literature in oral medicine and to explore the methods that have been used in this emerging field | book chapter | - | - | NA | - | describe the telehealth literature on oral medicine and to explore the methods that have been used in this emerging field |
| 50 | O. Gambino, | Italy  (2014) | present a Teledentistry system aimed to the Second Opinion task | System development and evaluation | - | - | NA | Present a teledentistry system for the Second opinion | a teledentistry system able to perform a video streaming, medical/radiological image sharing, and medical tele-reporting system |
| 51 | Rodrigo Mariño | Australia  (2014) | evaluation of the feasibility of a teledentistry model for teleconsultation and telediagnosis in Residential Aged Care Facilities | a Pilot Feasibility  Study | rural RACF  residents in Victoria, Australia. | Residential Aged Care Facilities (RACFs) in Victoria, Australia. | N=62 | - comparing remote assessments with traditional face-to-face oral examinations - using an intraoral camera by trained teledentistry assistants with the aim of screening residents for oral diseases and pathological conditions. | - teledentistry provide an innovative solution towards closing the service delivery gap in the provision of sustainable oral health care services to   underserviced populations (e.g., nursing homes, rural areas). |

Continue Table 2

|  | Author | country (year) | Aims | type of study | population | Study setting | Sample  size | Methodology | Main findings and implications |
| --- | --- | --- | --- | --- | --- | --- | --- | --- | --- |
| 52 | C. Torres-Pereira, | Brazil  (2013) | evaluate the applicability of telediagnosis in oral medicine by transmitting of clinical digital images via e-mail. | Feasibility | patients who came to the oral medicine clinic who needed a biopsy for a definitive diagnosis were invited to participate. | oral medicine clinic at the Federal University of Parana´ who needed a biopsy | n=60 | - Recording clinical history and oral lesion images with clinical electronic charts and a digital camera. - Email photo and information to two oral medicine consultants - formulating up to two diagnostic hypotheses for each case by consultant. - comparing diagnostic hypotheses to the gold standard (biopsy results) by means of percent agreement and kappa coefficient | - substantial agreement between the first consultant and the gold standard. kappa coefficients:   (k = 0.669)   - fair agreement between the second consultant and the gold standard. kappa coefficients:   (k = 0.574)   - Endorsing the use of e-mail and intraoral photos as a screening tool for oral diseases. |
| 53 | Saad Ahmed Khan | Malaysia  (2013) | summarize teledentistry in various dental applications has been reported. | Literature Review | - | - | NA | - | Description the use of teledentistry in various field of dentistry. |
| 54 | D. Dubovina, | Serbia  (2012) | Describe the procedure of posting teleconsultation requests by physicians, consultation seekers, and the procedure of responding to teleconsultation requests by physicians, | Case report | A patient with intraoral changes in the upper jaw fornix on the left side. Case2: A patient with a fistula in the area of tooth 22 | Department for Dental Diseases and Department of Oral Surgery, Dentistry Clinic, Faculty of Medicine in Priština - temporarily seated in Kosovska Mitrovica, | n=1 | Present a telemedicine system | The XPA3 Online teleconsultation system is a convenient and comfortable way for rapid and high-quality management of dentistry patients. |
| 55 | Blomstrand L | Sweden  (2012) | describe a telemedicine system as complement to traditional referrals in oral medicine | cross sectional | patients and  clinician | Department of Oral & Maxillofacial Surgery | n=10 | - Three male and 7 female patients were discussed during the telemedicine rounds - the photos can be viewed during telemedicine rounds and by the consultants at the hospital prior to a consultation. - The Use of Secure, interactive conferencing software is used | - using telemedicine in the dental health clinic could provide treatment without the need for referral to a consultant. - This telemedicine system allows patient care to be provided rapidly and more economically. |
| 56 | M. Bradley, | N. Ireland  (2010) | Feasibility the application of teledentistry in oral medicine in a Community Dental Service, | feasibility study | Patients with oral medicine conditions from all areas of Northern Ireland are referred by dentists and doctors  to a small number of specialist services: predominantly, the Regional Oral Medicine Consultant at the School of Dentistry, Belfast | School of Dentistry, Belfast | n=17 | - Develop a teledentistry prototype system - six month study to assess the   feasibility of using teledentistry to process oral medicine referrals to a Belfast hospital consultant. | - set up a prototype teledentistry system to support triage and management of oral medicine referrals - the teledentistry system demonstrated robust technical performance |
| 57 | James Fricton | USA  (2009) | discusses how innovative health information and communication technologies can improve access to oral health care through teledentistry | review | - | - | NA | - | describe applications of teledentistry |
| 58 | C. Torres-Pereira, | Brazil  (2008) | the feasibility of remote diagnosis in oral medicine | feasibility study | Patients with oral lesions | a public primary care unit 100 km from Curitiba in Parana´, Brazil | n=25 | - Producing Clinical electronic charts and images - Email information and image two oral medicine specialists - formulating up to two diagnostic hypotheses for each case by consultant. - comparing final diagnoses to the diagnoses of the remote clinicians by means of percent agreement and kappa coefficient | - fair agreement between clinicians. kappa coefficients: (k=0.28) - Remote diagnosis can be an effective alternative in the diagnosis of oral lesions - using two distant consultants improves diagnostic accuracy. |
| 59 | Le~ ao JC | England  (1999) | determine the possible acceptability of recording and transmitting clinical images of patients with orofacial diseases via the Internet, and thus develop the application of the use of this network in the remote diagnosis of oral disease. | feasibility | patients referred to the Oral Medicine unit, for the diagnosis and management of oral mucosal disease. | Oral Medicine unit of the Eastman Dental Institute for Oral Health Care Sciences, London, UK | n=20 | - capturing the digital images of oral mucosal lesion - store images on a personal computer and send them via the Internet to a remote location. - complete a self-administered questionnaire detailing their opinion of the use of an intra-oral camera by patients. - comparing the original and transmitted images by group of clinicians. | - from patients’ point of view, recording images of mouth very comfortable and useful in understanding their clinical problem - from clinicians’ point of view, there is not differentiate the original and transmitted image and they were able to accurately diagnose the patient s oral mucosal problems in 64% of the instances. |
