# Supplementary material for: Tele-dentistry, its trends, scope, and future framework in oral medicine; a scoping review during January 1999 to December 2021
Source: Arch Public Health. 2023 Jun 14;81:104. doi: 10.1186/s13690-023-01128-w (PMC10265557; doi:10.1186/s13690-023-01128-w)
Supplement: Supplementary file 2 — Additional file 2. [file 13690_2023_1128_MOESM2_ESM.docx]

**Appendix 2:**

**Table1 - Barriers of applying tele-dentistry services in oral medicine** *(scoping review 1991-2021)*

| **Main-themes** | **Sub-themes** | **Final codes** |
| --- | --- | --- |
| **Individual** | **Behavioral barriers** | Low motivation to use tele-dentistry services (60) |
|  |  | Non-adherence to the photography protocols(59) |
|  |  | Low level of patient information to communicate (23) |
|  |  | Uncomfortably feeling with tele-dentistry services (19) |
|  |  | Skepticism inherent for applying teleconsultations (37) |
|  |  | Ignoring cultural concerns (4) |
|  | **Lack of experience and skill** | Lack of digital literacy (25,45) |
|  |  | Inexperienced healthcare providers (60) |
|  |  | Lack of experience using tele-dentistry services (21) |
|  |  | Difficulty in taking intraoral photos by patient independently(11, 21) |
|  | **Human error** | Missing of patient data (24) |
|  |  | Inadequate documentation of patient records (8) |
| **Environmental** | **Inappropriate environmental conditions** | Lack of clinical environment for conducting teledentistry (1, 23, 55) |
|  |  | Noisy environment with limited privacy(36) |
|  |  | Poor illumination (11, 12, 23) |
| **Technical** | **Integration problems** | Problem in integration of tele-dentistry into clinical practice (3) |
|  | **Internet bandwidth problems** | Poor connectivity and problems in bandwidth (1, 23, 24) |
|  |  | Problem in data communication (28) |
|  | **Problems in data storing** | Problem in Uploading and storing all de-identifying patient data (23) |
|  |  | Challenge in large size files uploading (24) |
|  |  | Inaccuracy of database for the registered specialists (3) |
|  | **Security and confidentiality problems** | Security (26) |
|  |  | Confidentiality of data in photo messaging application (10, 36)  note: In data storage and transmission process |
|  | **Photo quality problems** | The effect of various color spectrum of phones on diagnostic efficiency (20) |
|  |  | Photo compressing (20) |
|  |  | Poor quality of image (24) |
|  | **Problems in real time interpretation** | Lack of real time interpretation for giving clinical opinion in urgent situation (44) |
|  |  | Challenge in accessibility to content (37) |
|  | **Accessibility problems** | Limitation in participants` number because of using Desktop conferencing (7) |
| **Regulation** | **legal and ethical issues** | Unknown legal and ethical issues (55) |
|  | **Prohibition of using some applications** | Prohibit use of a free messaging service in some countries (20) |
| **Organizational** | **Human resource problems** | Problems at the availability of human resource (1, 23) |
|  |  | Problems for training of human resource (1, 23) |
|  | **Administrative Challenges** | Administrative challenges (23) |
|  | **Financial Barriers** | Challenges in reimbursement (36) |
|  |  | Financial constraints (1) |
|  |  | Ignoring tele-dentistry cost-effectiveness and availability of adequate infrastructure (3, 9) |
|  | **Lack of guidelines** | Lack of standard methods for tele-dentistry in oral medicine (1) |
|  |  | Lack of a guideline for oral cavity photography (49) |
|  | **Lack of time management** | Lack of time for photography training (23) |
|  |  | Spend time to remote diagnosis in primary healthcare facilities (23, 24) |
| **Clinical** | **Problems in Patient examination** | Lack of tactile assessment (11, 13, 37, 53) |
|  |  | Telephone review appointments Oral Medicine without detailed examination (5) |
|  |  | Miss a lesion in the tele-dentistry mode (20) |
|  | **Making diagnosis problems** | The remote specialist opinion depends on missed imaging of the lesion and poor-quality images by the Community Health Workers (23, 26) |
|  |  | Inadequate interpretation of images by clinicians (44) |
|  | **Problems in performing**  **treatment plan** | Limitation of tele- management of dental emergencies to prescribing medication(70)  Restrictions on performing dental procedures in dental emergencies(70) |

**References:**

1. Torres-Pereira CC, Almeida Castro Morosini Id, Fonseca BB. Teledentistry and the distant diagnosis of oral mucosal disease. Teledentistry: Springer; 2015. p. 13-21.

2. Tella A, Olanloye O, Ibiyemi O. Potential of teledentistry in the delivery of oral health services in developing countries. Annals of Ibadan Postgraduate Medicine. 2019;17(2):115-23.

3. Al Mohaya MA, Almaziad MM, Al-Hamad KA, Mustafa M. Telemedicine among oral medicine practitioners during covid-19 pandemic and its future impact on the specialty. Risk Management and Healthcare Policy. 2021;14:4369.

4. Glick M, Greenberg MS, Lockhart PB, Challacombe SJ. Introduction to oral medicine and oral diagnosis: patient evaluation. Burket's Oral Medicine. 2021:1-18.

5. Macken J, Fortune F, Buchanan J. Remote telephone clinics in oral medicine: reflections on the place of virtual clinics in a specialty that relies so heavily on visual assessment. A note of caution. British Journal of Oral and Maxillofacial Surgery. 2021;59(5):605-8.

6. Flores APdC, Lazaro SA, Molina-Bastos CG, Guattini VLdO, Umpierre RN, Gonçalves MR, et al. Teledentistry in the diagnosis of oral lesions: A systematic review of the literature. Journal of the American Medical Informatics Association. 2020;27(7):1166-72.

7. Fricton J, Chen H. Using teledentistry to improve access to dental care for the underserved. Dental Clinics. 2009;53(3):537-48.

8. Tesfalul M, Littman-Quinn R, Antwi C, Ndlovu S, Motsepe D, Phuthego M, et al. Evaluating the potential impact of a mobile telemedicine system on coordination of specialty care for patients with complicated oral lesions in Botswana. Journal of the American Medical Informatics Association. 2016;23(e1):e142-e5.

9. Carrard V, Roxo Gonçalves M, Rodriguez Strey J, Pilz C, Martins M, Martins M, et al. Telediagnosis of oral lesions in primary care: The EstomatoNet Program. Oral diseases. 2018;24(6):1012-9.

10. Fonseca BB, Perdoncini NN, da Silva VC, Gueiros LAM, Carrard VC, Lemos Jr CA, et al. Telediagnosis of oral lesions using smartphone photography. Oral diseases. 2021.

11. Tenore G, Podda GM, La Torre G, Rocchetti F, Palaia G, Di Paolo C, et al. Evaluation of Patient’s Perception and Acceptance of Tele (oral) Medicine for Care during the COVID-19 Pandemic: A Cross-Sectional Pilot Survey. Applied Sciences. 2021;11(16):7443.

12. Lv N, Sun M, Polonowita A, Mei L, Guan G. Management of oral medicine emergencies during COVID-19: A study to develop practise guidelines. Journal of dental sciences. 2021;16(1):493-500.

13. Villa A, Sankar V, Shiboski C. Tele (oral) medicine: A new approach during the COVID‐19 crisis. Oral Diseases. 2020.

14. Estai M, Kanagasingam Y, Tennant M, Bunt S. A systematic review of the research evidence for the benefits of teledentistry. Journal of telemedicine and telecare. 2018;24(3):147-56.

15. Amtha R, Gunardi I, Astoeti TE, Roeslan MO. Satisfaction level of the oral medicine patients using teledentistry during the COVID-19 pandemic: a factor analysis. Journal of International Society of Preventive & Community Dentistry. 2021;11(4):414.

16. Meurer MI, Von Wangenheim A, Zimmermann C, Savaris A, Petrolini VA, Wagner HM. Launching a public statewide tele (oral) medicine service in Brazil during COVID-19 pandemic. Oral diseases. 2020.

17. Lopes MA, Santos-Silva AR, Vargas PA, Kowalski LP. Virtual assistance in oral medicine for prioritizing oral cancer diagnosis during the COVID-19 pandemic. Oral surgery, oral medicine, oral pathology and oral radiology. 2020;130(1):127.

18. Alves FA, Saunders D, Sandhu S, Xu Y, de Mendonça NF, Treister NS. Implication of COVID-19 in oral oncology practices in Brazil, Canada, and the United States. Oral diseases. 2021;27 Suppl 3:793-5.

19. Haron N, Zain RB, Ramanathan A, Abraham MT, Liew CS, Ng KG, et al. m-Health for early detection of oral cancer in low-and middle-income countries. Telemedicine and e-Health. 2020;26(3):278-85.

20. Vinayagamoorthy K, Acharya S, Kumar M, Pentapati KC, Acharya S. Efficacy of a remote screening model for oral potentially malignant disorders using a free messaging application: a diagnostic test for accuracy study. Australian Journal of Rural Health. 2019;27(2):170-6.

21. Zhou MX, Johnson EF, Arce K, Gruwell SF. Teledentistry in the management of a non‐Hodgkin's lymphoma manifesting as a gingival swelling: A case report. Special Care in Dentistry. 2022;42(1):86-90.

22. Muniz IdAF, Campos DES, Shinkai RSA, Trindade TGd, Cosme‐Trindade DC. Case report of oral mucosa garlic burn during COVID‐19 pandemic outbreak and role of teledentistry to manage oral health in an older adult woman. Special Care in Dentistry. 2021;41(5):639-43.

23. Birur P, Patrick S, Bajaj S, Raghavan S, Suresh A, Sunny SP, et al. A novel mobile-health approach to early diagnosis of oral cancer. The journal of contemporary dental practice. 2018;19(9):1122.

24. Birur NP, Gurushanth K, Patrick S, Sunny SP, Raghavan SA, Gurudath S, et al. Role of community health worker in a mobile health program for early detection of oral cancer. Indian Journal of Cancer. 2019;56(2):107.

25. Bradley M, Black P, Noble S, Thompson R, Lamey P. Application of teledentistry in oral medicine in a community dental service, N. Ireland. British dental journal. 2010;209(8):399-404.

26. Haron N, Zain RB, Nabillah WM, Saleh A, Kallarakkal TG, Ramanathan A, et al. Mobile phone imaging in low resource settings for early detection of oral cancer and concordance with clinical oral examination. Telemedicine and e-Health. 2017;23(3):192-9.

27. Khan SA, Omar H. Teledentistry in practice: literature review. Telemedicine and e-Health. 2013;19(7):565-7.

28. Tan SHX, Lee CKJ, Yong CW, Ding YY. Scoping review: Facilitators and barriers in the adoption of teledentistry among older adults. Gerodontology. 2021;38(4):351-65.

29. Arksey H, O'Malley L. Scoping studies: towards a methodological framework. International journal of social research methodology. 2005;8(1):19-32.

30. Peters M, Godfrey C, Khalil H, McInerney P, Soares C, Parker D. 2017 guidance for the conduct of JBI scoping reviews. Joana Briggs Inst Rev Man. 2017;13:141-6.

31. PRISMA. PRISMA for Scoping Reviews 2018 [Available from: <https://prisma-statement.org/Extensions/ScopingReviews>.

32. Kiger ME, Varpio L. Thematic analysis of qualitative data: AMEE Guide No. 131. Medical teacher. 2020;42(8):846-54.

33. Nowell LS, Norris JM, White DE, Moules NJ. Thematic analysis: Striving to meet the trustworthiness criteria. International journal of qualitative methods. 2017;16(1):1609406917733847.

34. Sanghvi R, Barrow S, Hullah E, De Souza M, Cook R, Siddik D. Paediatric dental-oral medicine clinic: management during COVID 19. Journal of Oral Medicine and Oral Surgery. 2021;27(1):18.

35. Pérez González A, Gallas Torreira M, Chamorro Petronacci CM, Pérez Sayáns M. Teledentistry: A New Approach in Dental Medicine. Enhanced Telemedicine and e-Health: Springer; 2021. p. 41-64.

36. Perdoncini NN, Schussel JL, Amenábar JM, Torres-Pereira CC. Use of smartphone video calls in the diagnosis of oral lesions: Teleconsultations between a specialist and patients assisted by a general dentist. The Journal of the American Dental Association. 2021;152(2):127-35.

37. Murthy V, Herbert C, Bains D, Escudier M, Carey B, Ormond M. Patient experience of virtual consultations in Oral Medicine during the COVID‐19 pandemic. Oral diseases. 2021.

38. Hayajneh Y. Systems and systems theory. Management for health care professionals series. Retrieved April. 2007;20:2010.

39. Dar-Odeh N, Babkair H, Alnazzawi A, Abu-Hammad S, Abu-Hammad A, Abu-Hammad O. Utilization of teledentistry in antimicrobial prescribing and diagnosis of infectious diseases during COVID-19 lockdown. European Journal of Dentistry. 2020;14(S 01):S20-S6.

40. Jampani N, Nutalapati R, Dontula B, Boyapati R. Applications of teledentistry: A literature review and update. Journal of International Society of Preventive & Community Dentistry. 2011;1(2):37.

41. Carrard VC, Martins MAT, Molina-Bastos CG, Gonçalves MR. WhatsApp: A telemedicine platform for facilitating remote oral medicine consultation and improving clinical examinations—some considerations. Oral surgery, oral medicine, oral pathology and oral radiology. 2017;123(3):408.

42. Birur PN, Sunny SP, Jena S, Kandasarma U, Raghavan S, Ramaswamy B, et al. Mobile health application for remote oral cancer surveillance. The Journal of the American Dental Association. 2015;146(12):886-94.

43. Georgakopoulou EA. Digitally aided telemedicine during the SARS‐CoV‐2 pandemic to screen oral medicine emergencies. Oral Diseases. 2020.

44. Leao JC, Porter SR. Telediagnosis of oral disease. Braz Dent J. 1999;10(1):47-53.

45. Ghanbarzadegan A, Bastani P, Luzzi L, Brennan D. Inequalities in utilization and provision of dental services: a scoping review. Systematic reviews. 2021;10(1):1-11.

46. Tingle J. Improving the quality of patient-based information. HEALTH CARE RISK REPORT. 2002;8:10-1.

47. Mohammed SA, Yusof MM. Towards an evaluation framework for information quality management (IQM) practices for health information systems–evaluation criteria for effective IQM practices. Journal of evaluation in clinical practice. 2013;19(2):379-87.

48. Cabitza F, Batini C. Information Quality in Healthcare. In: Batini C, Scannapieco M, editors. Data and Information Quality: Dimensions, Principles and Techniques. Cham: Springer International Publishing; 2016. p. 403-19.

49. Lin I, Datta M, Laronde DM, Rosin MP, Chan B. Intraoral Photography Recommendations for Remote Risk Assessment and Monitoring of Oral Mucosal Lesions. Elsevier; 2021. p. 384-9.

50. Batra P, Tagra H, Katyal S. Artificial Intelligence in Teledentistry. Discoveries. 2022;10(3).

51. Patil S, Albogami S, Hosmani J, Mujoo S, Kamil MA, Mansour MA, et al. Artificial intelligence in the diagnosis of oral diseases: applications and pitfalls. Diagnostics. 2022;12(5):1029.

52. Villa A, Sankar V, Shazib MA, Ramos D, Veluppillai P, Wu A, et al. Patient and providers' satisfaction with tele (oral) medicine during the COVID‐19 pandemic. Oral diseases. 2020.

53. Gilligan GM, Piemonte ED, Lazos JP, Panico RL. In reply to the letter to the editor" Tele (oral) medicine: A new approach during the COVID-19 crisis". 2020.

54. Kumar KS, Venkitachalam R, Varma B, Nair PK, Shanmugham AM, Jose R. Oral medicine practice during COVID-19: A scoping review. Journal of Indian Academy of Oral Medicine and Radiology. 2021;33(2):215.

55. Emodi-Perlman A, Eli I. One year into the COVID-19 pandemic–temporomandibular disorders and bruxism: What we have learned and what we can do to improve our manner of treatment. Dental and Medical Problems. 2021;58(2):215-8.

56. Deshpande S, Patil D, Dhokar A, Bhanushali P, Katge F. Teledentistry: A Boon Amidst COVID-19 Lockdown—A Narrative Review. International Journal of Telemedicine and Applications. 2021;2021.

57. Roxo-Gonçalves M, Trevizani Martins MA, Martins MD, Aita Schmitz CA, Dal Moro RG, D'Avila OP, et al. Perceived usability of a store and forward telehealth platform for diagnosis and management of oral mucosal lesions: A cross-sectional study. PloS one. 2020;15(6):e0233572.

58. Torres-Pereira CC, Morosini IdAC, Possebon RS, Giovanini AF, Bortoluzzi MC, Leao JC, et al. Teledentistry: distant diagnosis of oral disease using e-mails. Telemedicine and e-Health. 2013;19(2):117-21.

59. Estai M, Kanagasingam Y, Xiao D, Vignarajan J, Bunt S, Kruger E, et al. End-user acceptance of a cloud-based teledentistry system and Android phone app for remote screening for oral diseases. Journal of telemedicine and telecare. 2017;23(1):44-52.

60. Roxo-Gonçalves M, Strey JR, Bavaresco CS, Martins MAT, Romanini J, Pilz C, et al. Teledentistry: A tool to promote continuing education actions on oral medicine for primary healthcare professionals. Telemedicine and e-Health. 2017;23(4):327-33.

61. Maret D, Warnakulasuriya S, Herbault-Barres B, Savall F, Vigarios E. Telemedicine contributing to an incidental finding of a premalignant lesion. Oral Oncology. 2021;118:105331.

62. Torres-Pereira C, Possebon RS, Simões A, Bortoluzzi MC, Leao JC, Giovanini AF, et al. Email for distance diagnosis of oral diseases: a preliminary study of teledentistry. Journal of Telemedicine and Telecare. 2008;14(8):435-8.

63. Inquimbert C, Hirata-Tsuchiya S, Yoshii S, Molinari N, Nogue E, Roy C, et al. Concordance study between regular face-to-face dental diagnosis and dental telediagnosis using fluorescence. Journal of Telemedicine and Telecare. 2021;27(8):509-17.

64. Maret D, Peters OA, Auria J-P, Savall F, Vigarios E. Smartphone oral self-photography in teledentistry: Recommendations for the patient. Journal of telemedicine and telecare. 2021:1357633X211028513.

65. Vetchaporn S, Rangsri W, Ittichaicharoen J, Rungsiyakull P. Validity and Reliability of Intraoral Camera with Fluorescent Aids for Oral Potentially Malignant Disorders Screening in Teledentistry. International Journal of Dentistry. 2021;2021.

66. Mariño R, Hopcraft M, Tonmukayakul U, Manton D, Marwaha P, Stanieri A, et al. Teleconsultation/telediagnosis using teledentistry technology: a pilot feasibility study. Int J Adv Life Sci. 2014;6(3-4):291-9.

67. Queyroux A, Saricassapian B, Herzog D, Müller K, Herafa I, Ducoux D, et al. Accuracy of teledentistry for diagnosing dental pathology using direct examination as a gold standard: results of the tel-e-dent study of older adults living in nursing homes. Journal of the American Medical Directors Association. 2017;18(6):528-32.

68. Petruzzi M, De Benedittis M. WhatsApp: a telemedicine platform for facilitating remote oral medicine consultation and improving clinical examinations. Oral surgery, oral medicine, oral pathology and oral radiology. 2016;121(3):248-54.

69. Araújo ALD, do Amaral-Silva GK, Pérez-de-Oliveira ME, Gallagher KPD, López de Cáceres CVB, Roza ALOC, et al. Fully digital pathology laboratory routine and remote reporting of oral and maxillofacial diagnosis during the COVID-19 pandemic: a validation study. Virchows Archiv. 2021;479:585-95.

70. Binaisse P, Dehours E, Bodéré C, Chevalier V, Le Fur Bonnabesse A. Dental emergencies at sea: A study in the French maritime TeleMedical Assistance Service. Journal of Telemedicine and Telecare. 2020;26(5):285-93.

71. Blomstrand L, Sand LP, Gullbrandsson L, Eklund B, Kildal M, Hirsch J-M. Telemedicine—A complement to traditional referrals in oral medicine. Telemedicine and e-Health. 2012;18(7):549-53.

72. Chawla J, Yerragudi N. Could Tele-Oral medicine help in early diagnosis of oral cancer? Journal of Oral Medicine and Oral Surgery. 2021;27(4):48.

73. Dubovina D, Mihailovic B, Vujicic B, Tabakovic S, Matvijenko V, Zivkovic D. Teleconsultation in dentistry using the XPA3 online system: Case Report. Acta Facultatis Medicae Naissensis. 2012;29(2):93.
